# Dynamic Outlier Slicing Allows Broader Exploration of Adaptive Divergence: A Comparison of Individual Genome and Pool‐Seq Data Linked to Humic Adaptation in Perch

**DOI:** 10.1111/mec.17659

**Published:** 2025-01-23

**Authors:** María‐Eugenia López, Mikhail Ozerov, Lilian Pukk, Kristina Noreikiene, Riho Gross, Anti Vasemägi

**Affiliations:** ^1^ Institute of Freshwater Research, Department of Aquatic Resources (SLU Aqua) Swedish University of Agricultural Sciences Drottningholm Sweden; ^2^ Biodiversity Unit University of Turku Turku Finland; ^3^ Chair of Aquaculture Estonian University of Life Sciences Tartu Estonia; ^4^ Institute of Biosciences, Life Sciences Center Vilnius University Vilnius Lithuania

**Keywords:** Humic lakes, Outliers, selection signatures, WGS

## Abstract

How genetic variation contributes to adaptation at different environments is a central focus in evolutionary biology. However, most free‐living species still lack a comprehensive understanding of the primary molecular mechanisms of adaptation. Here, we characterised the targets of selection associated with drastically different aquatic environments—humic and clear water—in the common freshwater fish, Eurasian perch ( *Perca fluviatilis* ). By using whole‐genome sequencing (WGS) on a large population dataset (*n* = 42 populations) and analysing 873,788 SNPs, our primary aim was to uncover novel and confirm known footprints of selection. We compared individual and pooled WGS, and developed a novel approach, termed dynamic outlier slicing, to assess how the choice of outlier‐calling stringency influences functional and Gene Ontology (GO) enrichment. By integrating genome‐environment association (GEA) analysis with allele frequency‐based approaches, we estimated composite selection signals (CSS) and identified 2679 outlier SNPs distributed across 324 genomic regions, involving 468 genes. Dynamic outlier slicing identified robust enrichment signals in five annotation categories (upstream, downstream, synonymous, 5′UTR and 3′UTR) highlighting the crucial role of regulatory elements in adaptive evolution. Furthermore, GO analyses revealed strong enrichment of molecular functions associated with gated channel activity, transmembrane transporter activity and ion channel activity, emphasising the importance of osmoregulation and ion balance maintenance. Our findings demonstrate that despite substantial random drift and divergence, WGS of high number of population pools enabled the identification of strong selection signals associated with adaptation to both humic and clear water environments, providing robust evidence of widespread adaptation. We anticipate that the dynamic outlier slicing method we developed will enable a more thorough exploration of adaptive divergence across a diverse range of species.

## 1 Introduction

Understanding the genetic mechanisms underlying adaptation is one of the fundamental goals of population genetics and evolutionary biology (Allendorf and Ryman 2002; Bernatchez 2016). Advances in high‐throughput sequencing technologies have significantly enhanced our capacity to explore these mechanisms, particularly through the application of hitchhiking mapping (Harr, Kauer, and Schlötterer 2002), also known as ‘genome scan’ approaches (Rellstab et al. 2015; Ahrens et al. 2018). Whole‐genome re‐sequencing provides the highest resolution for detecting both neutral and putative adaptive regions within a species' genome. Compared to less densely sampled SNP datasets, this approach has enabled the identification of fine‐scale peaks of genetic differentiation between populations, as well as strong association with phenotypes that were undetected using reduced‐representation methods (see e.g., (a) Aguillon, Walsh, and Lovette (2021) vs. Aguillon et al. (2018); (b) Campagna et al. (2017) vs. Campagna et al. (2015) (c) Clucas, Kerr et al. (2019) vs. Clucas, Lou et al. (2019); and (d) Szarmach et al. (2021)). However, reliable genetic insights often require a large sample sizes, making individual whole‐genome sequencing (WGS) cost‐prohibitive. Pooling individuals offers a cost‐effective alternative for SNP discovery and allele frequency estimation (Futschik and Schlötterer 2010; Gautier et al. 2013). This cost‐efficiency of pooled sequencing (pool‐seq) has driven its widespread adoption in investigating the genetic basis of complex traits (e.g., Cheeseman et al. 2015), identifying loci linked to local adaptation (e.g., Giska et al. 2022), and discerning genomic regions under selection during domestication (e.g., Rubin et al. 2010), among other applications.

In the exploration of genomic regions shaped by adaptation, researchers often compare populations living in different environments to detect highly differentiated genetic regions among them (via allele frequency differentiation or outlier methods), and identify correlations between allele frequencies (AF) and environmental variables (genome‐environment association, GEA) (Hoban et al. 2016; Ahrens et al. 2018). However, accurately modelling the null distribution and calculating precise *p*‐values for empirical datasets is challenging due to factors such as population structure, sample design (Lotterhos and Whitlock 2015), recombination rates (Booker, Yeaman, and Whitlock 2020) and approach can enhance the robustness (Grossman et al. 2010; Utsunomiya et al. 2013; Ma et al. 2015; Lotterhos et al. 2017). Furthermore, despite substantial algorithmic enhancements, one of the oft‐discussed questions regarding genome scans pertains to the determination of which statistical threshold should be used to call a locus an outlier, significantly deviating from the neutral expectations (François et al. 2016). Across and even within studies, various significance thresholds are often employed to identify selection signatures. These thresholds often encompass the upper percentiles of the observed distribution, typically ranging from the top 5% to 0.1%, or rely on statistical measures such as *p*‐values, *q*‐values, standard deviations or other relevant statistics.

Thus, determining the most appropriate significance thresholds can be challenging and is often arbitrary, given the apparent trade‐offs between overly stringent or relaxed criteria associated with potential false negative and positive detections (Whitlock and Lotterhos 2015; Whitlock and Lotterhos 2015). Here, we present a complementary approach using to assess the non‐random distribution of SNP categories and Gene Ontology (GO) terms across different levels of outlier stringency levels. Since the true footprints of selection are expected to be enriched for certain type of variants (e.g., regulatory, missense) more than the others (e.g., intergenic, intronic) and correspond to specific molecular function (MM), cellular component (CC) and biological processes (BP) linked to the physiological process of adaptation, we tested how different thresholds of calling a set of loci as outliers influence their functional enrichment. We predicted that the use of small number of outliers with very stringent thresholds (high level of false negatives) would result in a reduced power to observe significant functional enrichment. In contrast, overly relaxed outlier thresholds with high number of false positives, is expected to weaken the true signal of functional enrichment. Thus, by screening across different outlier thresholds, we expect to obtain new functional information on the interplay between putative false negative and positive detections. We call this procedure ‘dynamic outlier slicing’ to reflect its explorative nature. This approach allows us to systematically explore the impacts of varying outlier thresholds on non‐random distribution of variants shaped by divergent selection and functional enrichment of genes important for adaptation. Furthermore, it is applicable to any dataset that ranks loci based on the strength of evidence for selection or non‐neutrality, whether derived from individual or pooled data.

In aquatic environments, fish are found in almost all habitat types, including those that pose extreme survival challenges (Wang and Guo 2019), such as caves (Proudlove et al. 2010; Soares and Niemiller 2013), high hydrogen sulphide concentrations (Plath et al. 2007; Riesch, Plath, and Schlupp 2010), hypoxia (Yang et al. 2021), hypersaline and hyperalkaline water (Tong et al. 2017; Xu et al. 2017; Tong and Li 2020). Among these extreme environments are also dystrophic lakes, which are characterised by high content of humic substances, low pH values, nutrient‐poor and brown‐coloured waters (Kalinowska et al. 2021; Karpowicz et al. 2023). These characteristics act as limiting factors for the species richness, diversity and abundance of zooplankton and fish communities in temperate zone (Arvola and Kankaala 1989; Finstad et al. 2014; Kalinowska et al. 2021). The dark brownish hue of water in these lakes is primarily attributed to the presence of dissolved organic matter (DOM), originating from surrounding forests and peat bogs as well as the decomposition of aquatic plant materials and bacteria (Wetzel 2001; Stedmon, Markager, and Bro 2003). DOM is typically quantified as dissolved organic carbon (DOC; Wood, Al‐Reasi, and Smith 2011), and it significantly regulates the carbon and energy cycle of inland waters and plays a pivotal role in shaping aquatic ecosystems, impacting their biological, chemical, and physical characteristics (Battin et al. 2009). Over recent years, terrestrial loads of dissolved organic carbon have increased in lakes and rivers across various regions, a process known as brownification (Evans, Monteith, and Cooper 2005; Evans et al. 2006; Williamson et al. 2015; Meyer‐Jacob et al. 2019). This process has the potential to bring about significant alterations in the chemical, physical, and biological attributes of aquatic ecosystems (Brothers et al. 2014; Jones and Lennon 2015; Hedström et al. 2017), leading to changes in both planktonic and benthic primary production due to strong changes in light and nutrient availability (Kazanjian et al. 2021).

Eurasian perch ( *Perca fluviatilis* ) is one of the few fish species capable of thriving in acidic and humic conditions of northern latitude lakes (Hesthagen et al. 1992; Bertolo and Magnan 2007; Rask et al. 2014; Vasemägi et al. 2023). Yet, a comprehensive understanding of the genetic basis and molecular mechanisms that enable Eurasian perch to thrive in these conditions is still lacking. Recently, Ozerov et al. (2022) provided initial insights on footprint of selection associated to adaptation to the humic environment by analysing full genomes of 32 individuals. This study revealed hundreds of genomic regions scattered across the genome shaped by divergent selection, and pinpointed the importance of plasma membrane and ion transportation processes in humic adaptation. Furthermore, the enrichment of outlier variants in regulatory regions indicated the importance of regulatory elements in humic adaptation (Ozerov et al. 2022). However, because this study analysed only a single specimen per lake, the allele frequency estimates for individual populations could not be accurately estimated. Therefore, the previously identified footprints of selection likely represent only the most drastic differences between habitats (Ozerov et al. 2022).

In this study, we conducted a more comprehensive investigation using pooled WGS (pool‐seq) across a larger number of populations (*n* = 42) sampled from lakes with extremely dark and clear waters. Our specific objectives include: (1) to compare individual and pooled WGS datasets to characterise population genetic diversity and structure; (2) to identify outlier SNPs and genomic regions likely subjected to selection; (3) to develop and test a dynamic outlier slicing approach to assess how varying levels of stringency in outlier calling influence functional and GO enrichment analyses; and (4) to identify the most promising candidate genes and genomic regions involved in humic and clear water adaptation.

## 2 Materials and Methods

### 2.1 Sample Collection and Whole Genome Sequencing

**FIGURE 1:**
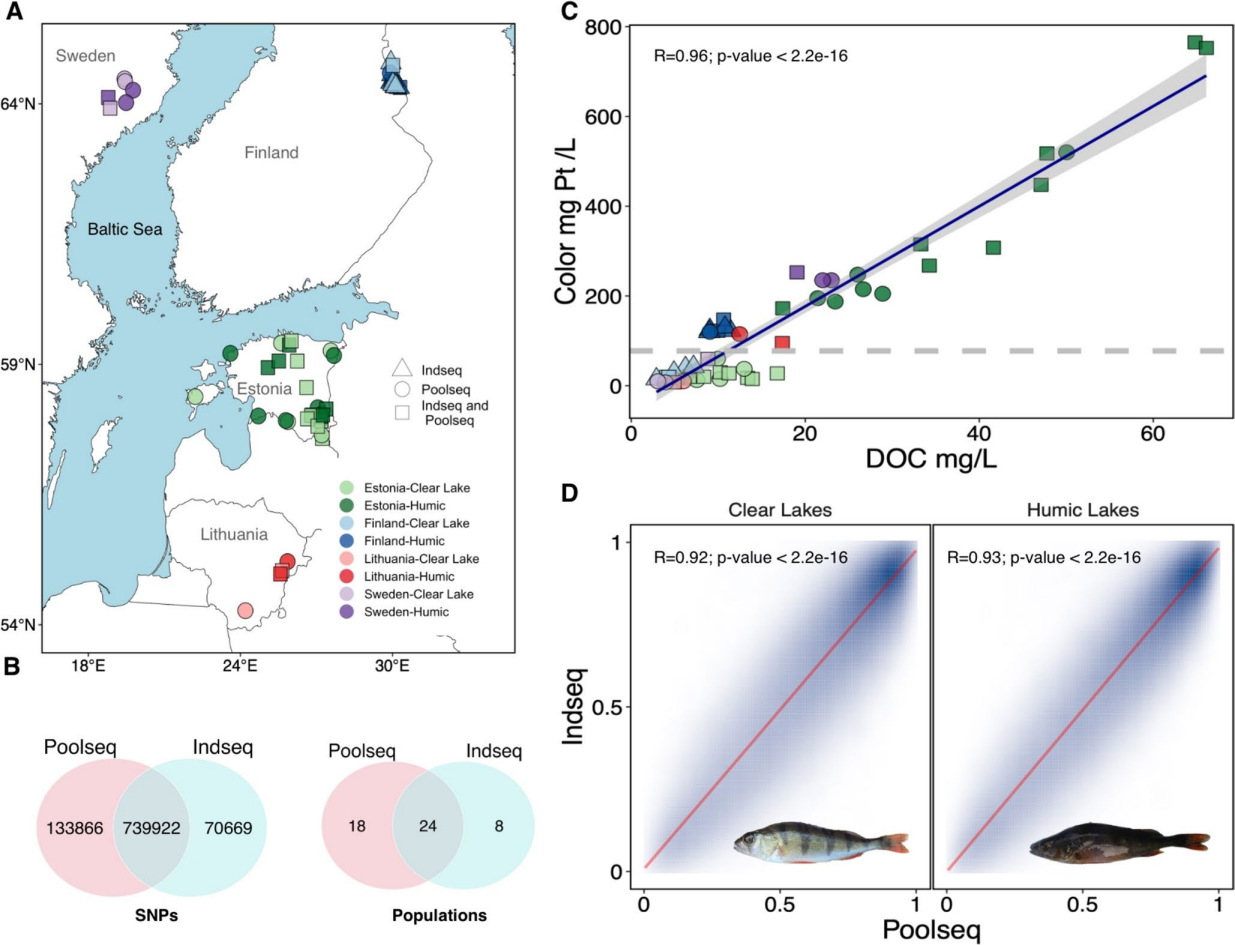
Geographic origins and overview of datasets for the study populations. (A) Map illustrating the geographic origins of all samples analysed. Colours denote countries: Greens for Estonia, blues for Finland, reds for Lithuania and purples for Sweden. Symbols represent sequencing techniques: Circles indicate populations sequenced using pooled sequencing (pool‐seq), triangles indicate individual sequencing (ind‐seq), and squares denote populations analysed with both techniques. (B) Venn diagrams depicting the overlap of SNPs and populations for ind‐seq and pool‐seq. (C) Correlation between dissolved organic content (DOC) in mg/L and coloration in mg Pt/L. The dashed line represents the midpoint value between the highest coloration in clear water lakes and the lowest coloration in the humic lakes. (D) Allele frequency correlations between ind‐seq and pool‐seq for populations in clear and humic lakes.

A total of 42 populations collected from four countries (Sweden, Finland, Estonia, Lithuania) were studied, comprising 22 populations from humic lakes and 20 populations from clear‐water lakes (Figure 1A, Table 1). Lakes were selected based on drastic differences in water colour while maintaining geographical proximity between lake types. Priority was given to lakes without outflows or, alternatively, those with migration barriers when outflows were present, to minimise the potential impact of gene flow on our inferences. Although our study design accounts for geographic proximity and contrasting environmental conditions, it is not a strictly paired experimental design. Instead, we evaluated differences between humic and clear water lakes population as aggregated groups. This approach enhances the power of detecting loci under selection while minimising the effect of population structuring (De Mita et al. 2013; Whitlock and Lotterhos 2015; Hoban et al. 2016).

**TABLE 1:** Summary information on studied populations, including country of origin, lake type (H—Humic, C—Clear‐water), pool sample size, latitude, longitude, dissolved organic carbon concentration (DOC, mg L^−1^), coloration (mgPt L^−1^) and sequencing depth.

ID	Country	Lake type	Lake name	N	Latitude	Longitude	DOC	Coloration	Sequencing depth
EAAK	Estonia	H	Akste järv	10	58.17	27.05	28.89	205	37.4
EANT^a^	Estonia	C	Äntu Valgejärv	40	59.06	26.24	13.38	17.5	42.5
EHEI^a^	Estonia	H	Heisri Mustjärv	41	58.02	26.83	33.28	315	62.2
EHIN^a^	Estonia	C	Hino järv	40	57.58	27.23	13.91	15	52.6
EHKI	Estonia	H	Holvandi Kivijärv	40	58.04	27.20	50.04	520	60.6
EKAH	Estonia	C	Kurtna Ahnejärv	10	59.26	27.56	6.25	17.5	44.4
EKAR	Estonia	C	Karujärv	8	58.38	22.22	10.2	15	28.2
EKIS	Estonia	C	Kisõjärv	40	57.64	27.21	12.98	37.5	40.7
EKUU^a^	Estonia	H	Kuulma järv	40	57.96	27.16	47.1	447.5	72.2
EKVA	Estonia	C	Koorküla Valgjärv	28	57.90	25.87	7.54	12.5	27.5
ELAS	Estonia	H	Lasa järv	16	57.92	25.79	26.03	247.5	33.0
ELOO^a^	Estonia	H	Loosalu järv	40	58.94	25.08	17.41	172.5	52.0
EMAH	Estonia	C	Mähuste järv	12	59.41	25.61	9.92	60	24.0
EMAT^a^	Estonia	H	Matsimäe Pühajärv	40	59.06	25.51	41.63	307.5	68.8
EMEE^a^	Estonia	H	Meelva järv	40	58.14	27.39	47.77	517.5	59.9
ENIG	Estonia	H	Nigula järv	12	58.01	24.71	26.66	215	17.1
EPAI^a^	Estonia	C	Paidra järv	40	57.91	27.19	10.23	30	79.8
EPAL	Estonia	H	Peraküla Allikajärv	11	59.21	23.61	23.44	187.5	37.4
EPAR^a^	Estonia	H	Partsi Saarjärv	40	58.00	27.17	64.8	765	62.7
EPII^a^	Estonia	C	Piigandi järv	40	58.02	26.79	8.34	20	32.4
EPUH	Estonia	H	Puhatu järv	12	59.17	27.69	21.43	195	33.8
ESAA^a^	Estonia	C	Saadjärv	40	58.55	26.61	11.24	27.5	53.1
EUDR^a^	Estonia	H	Udriku Suurjärv	40	59.37	25.92	34.26	267.5	54.6
EUIA^a^	Estonia	C	Uiakatsi järv	22	57.95	26.64	6.68	20	43.9
EVER^a^	Estonia	C	Kasaritsa Verijärv	40	57.81	27.05	16.78	27.5	68.9
EVII^a^	Estonia	C	Viitna Pikkjärv	40	59.45	26.01	5.27	17.5	61.2
EVIR^a^	Estonia	H	Virosi järv	31	58.03	27.26	66.1	752.5	58.2
FIMU^a^	Finland	H	Isomustalampi	10	64.31	30.29	9.97	122.5	68.1
FIVA^a^	Finland	C	Iso‐Valkeainen	10	64.70	30.00	4.07	20	59.9
FKAL^a^	Finland	H	Kalletomanlampi	8	64.36	29.97	10.62	147.5	63.9
FLLA	Finland	H	Lehtolampi	10	64.59	29.90	9.04	120	57.0
FPPE^a^	Finland	C	Pitkän‐Perjantai	8	64.74	30.01	4.32	20	55.4
LTGIR	Lithuania	H	Girutiškis	22	55.21	25.86	12.47	115	43.9
LTILG	Lithuania	C	Ilgis	25	54.27	24.20	5.99	10	48.5
LTLEI^a^	Lithuania	C	Leikštikas	28	55.04	25.65	4.93	7.5	56.5
LTPUR^a^	Lithuania	H	Purvynas	19	55.03	25.63	17.37	95	45.8
SWABO	Sweden	C	Aborrträsk	29	64.48	19.43	3.925	7.5	34.2
SWNB^a^	Sweden	H	Nedre Björntjärnen	20	64.12	18.78	19.05	252.5	38.0
SWOSK	Sweden	C	Östra Skärträsket	59	64.43	19.45	3.05	10	46.3
SWSNO^a^	Sweden	C	Snotterntjärnen	10	63.92	18.86	8.80	60	32.6
SWSTO	Sweden	H	Stortjärnen	23	64.26	19.76	23	235	30.1
SWSTR	Sweden	H	Struptjärnen	26	64.02	19.49	22	235	44.6

^a^ Indicate populations that were also included in the study by Ozerov et al. (2022), where WGS was performed on a single individual per lake.

Genomic DNA from each individual fish was extracted from tissue samples using the NucleoSpin Tissue kit (Macherey‐Nagel) following the manufacturer's protocol. For each population, DNA from individuals were pooled equimolarly, with pool sizes ranging from 8 to 59 individuals per pool (mean 26.7, 25–75th percentile = 11.8–40.0). Whole‐genome sequencing (WGS) was subsequently performed on these pooled population samples. Paired‐end libraries were prepared for each pool using a TruSeq PCR‐free kit (Illumina). The libraries were sequenced using an Illumina NovaSeq 6000 using paired‐end sequencing (2 × 150‐bp read length with 8‐bp index) at the Science for Life Laboratory (SciLifeLab), Uppsala, Sweden.

### 2.2 Read Quality and Variant Calling

The quality of the sequence data was assessed using FastQC v.0.11.8 (http://www.bioinformatics.babraham.ac.uk/projects/fastqc/). Short (< 60 bp) and low quality reads (average quality score < 25 in sliding window of 5 bp), poly‐G tails and Illumina adaptors were trimmed with fastp v.0.20 (Chen et al. 2018) using the following parameters: ‐g ‐w 12 ‐r ‐W 5 ‐M 25 ‐‐trim_front1 9 ‐‐trim_front2 9 ‐‐trim_tail1 2 ‐‐trim_tail2 2 ‐l 60. Filtered reads from each pool were aligned to the Eurasian perch reference genome (GenBank version: GCA_010015445.1) using Bowtie2 v.2.4.4 (Langmead et al. 2009). Default parameters were applied, with the exception of the modified score minimum threshold (−‐score‐min L, ‐0.3, ‐0.3) and the maximum fragment length for valid paired‐end alignments (−X 700).

The variant calling was carried out using two pipelines as performed in Ozerov et al. (2022), briefly: (1) The SAMtools v.1.10 (Li 2011) pipeline was applied to the aligned and sorted BAM files, then bcftools was applied to perform the variant calling with parameters set as: samtools mpileup ‐uIg ‐t DP,AD,INFO/AD,ADF,ADR,SP ‐q 20. (2) HaplotypeCaller subroutine from gatk v.n 4.1.4.1 (McKenna et al. 2010) which was applied to the BAM files to generate single‐sample GVCF files using the following parameters: ‐ERC GVCF ‐‐minimum‐mapping‐quality 20 ‐mbq 13 ‐‐indel‐size‐to‐eliminate‐in‐ref‐model 12 ‐G AS_StandardAnnotation. The GenomicsDBImport tools was used to import the GVCF files into GenomicsDB. Finally, a final calling of the consensus genotypes was performed with GenotypeGVCFs. Variants discovered by both pipelines were further filtered using vcftools v.0.1.15 (Danecek et al. 2011) as follows: ‐‐max‐meanDP 66 ‐‐min‐meanDP 10 ‐‐max‐missing 1 ‐‐mac 2 ‐‐min‐alleles 2 ‐‐max‐alleles 2 ‐‐minQ 30. Furthermore, variants occurring in repetitive genomic regions were excluded with ‐exclude‐bed parameter using positions of low complexity regions in perch genome. Finally, we retained 1,635,970 variants consistently called by both pipelines. The bcftools module was employed to generate an *mpileup* format file using SNPs positions. This file was then processed using PoPoolation2 (Kofler, Pandey, and Schlötterer 2011) to obtain a sync format file (mpileup2sync.pl), which contains the synchronised variant information across multiple populations. We further applied a minor allele frequency (MAF) threshold of 5% filter, resulting in a final dataset of 873,788 SNPs, distributed across 24 chromosomes, with a small portion located in unplaced scaffolds (Table S1).

The functional annotation of the SNPs was carried with SnpEff v.5.0 (Cingolani et al. 2012), employing the SnpEff database generated from the Eurasian perch reference genome sequence and its corresponding annotation file (NCBI: GCA_010015445.1). Furthermore, we identified the orthologous genes counterparts in the human and zebrafish genomes using RENTREZ (Winter 2017).

### 2.3 Comparison of Individual Versus Pooled WGS Datasets

One of the objectives of our study was to assess the performance of individual and pooled WGS approaches for detection of footprints of selection. To achieve this, we conducted a comparative analysis between a previously published dataset (Ozerov et al. 2022) and a new dataset comprising 42 populations generated for this study. The individual WGS dataset, referred to as ‘ind‐seq’ included 32 genomes sampled from 16 humic and 16 clear‐water lakes in Northern Europe, with a single individual per lake (Figure 1A). This dataset consists of 810,591 SNPs, a more detailed description can be found in Ozerov et al. (2022). The pooled dataset, henceforth referred to as ‘pool‐seq’ comprised the 42 populations and 873,788 SNPs, as mentioned earlier. Altogether, 24 populations (lakes) and 739,922 SNPs overlapped between these two datasets (Figure 1B). Individuals analysed by Ozerov et al. (2022) were also included for pool‐seq, with one individual overlapping in each of 24 shared populations.

### 2.4 Genetic Population Structure, Differentiation and Diversity

The allele frequency for each pool was calculated using the *calfreq* function within the popoolation2helper tool, which uses the synchronised data format (sync file; https://github.com/Yiguan/popoolation2helper). Diversity statistics, including segregating sites (S), nucleotide diversity (π) and Watterson's θ were computed using NPStats v1 (Ferretti, Ramos‐Onsins, and Pérez‐Enciso 2013), with parameters ‐l 50,000 –mincov 4 and –maxcov 500, where ‐l is the window length in bases, mincov minimum coverage and maxcov maximum coverage. Heterozygosity estimates within populations were calculated using the *compute.fstats* function implemented in PoolFstat v2.1.2 (Gautier et al. 2022), which employs the formula H = 1‐Q1. Here, Q1 represents the probability of identity in state (IIS or AIS for Alike‐In‐State) within a population. A principal component analysis (PCA) was performed using AF and the *prcomp* function within the R v.4.3.1 environment to visualise the relationships among the studied populations. To quantify population differentiation between pools, pairwise fixation indices (*F*_ST_) were calculated using *compute.pairwiseFST* function in PoolFstat v.2.1.2 in R (Gautier et al. 2022).

### 2.5 Genetic Signatures of Selection

Combining diverse methodologies for detecting loci deviating from neutral expectations is expected to facilitate identification of genomic regions potentially under selection (Vasemägi and Primmer 2005; Rellstab et al. 2015; François et al. 2016; Dalongeville et al. 2018). Accordingly, we employed two different strategies for detection of loci potentially associated with humic adaptation by using: (1) allele frequency differentiation among populations and (2) association between AF and environmental variables (genotype‐environment association, GEA). The genetic divergence between humic and clear‐water pools was assessed by first calculating AF separately for each population. Then, the mean allele frequency for humic and clear‐water groups were calculated, followed by determining the absolute allele frequency difference (|AFD|) between humic and clear‐water populations.

For the exploration of genetic variants associated with environmental variables, we performed a redundancy analysis (RDA). RDA is one of the best performing GEA approaches and exhibits low false‐positive rates (Capblancq and Forester 2021). RDA was carried out with the R package vegan v.2.6–4 (Oksanen et al. 2018). Two indicators that characterised the presence of DOM and the visual conditions within lakes were used: DOC (mg L^−1^) and coloration (mg Pt L^−1^). Despite high correlation between these two parameters (Figure 1C), in addition to DOC, water coloration is influenced by additional factors, such as iron concentration in the water (Maloney et al. 2005; Weyhenmeyer, Prairie, and Tranvik 2014; Lei, Thompson, and McDonald 2020). For RDA, we accounted for the influence of genetic and spatial structure by incorporating the principal component (PC) 1 loadings extracted form PCA on intergenic SNPs (Figure S1), which explained a significant proportion of the variation (10%) and captured broad‐scale spatial patterns. Additionally, we included the geographic coordinates of the lakes to further account for spatial structure.

### 2.6 Composite Selection Signals

Recent studies have demonstrated that employing composite measures of selection significantly improves the signal‐to‐noise ratio and increases the power of genome scans for selection signatures (Ma et al. 2015; Lotterhos et al. 2017) compared to using overlaps of single statistics. Here, we combined AFD and RDA to use the composite selection signals (CSS) approach implemented in R package MINOTAUR v.0.0.1 (Verity et al. 2017). Raw statistics were converted to fractional ranks and then transformed into z‐scores using the *CSS* function. Genome‐wide *p*‐values were computed using the *stat_to_pvalue* function. The CSS *p*‐values were then transformed to the corresponding *q*‐values using the *p.adjust* R function and the Benjamini and Hochberg method (Benjamini and Hochberg 1995). To further explore genomic regions potentially under selection, we defined SNPs as putative outliers with *q*‐values lower than 0.05. This criterion was applied to both ind‐seq and pool‐seq datasets. In the study conducted by Ozerov et al. (2022), candidate SNPs under selection were defined as those detected by at least two of the three methods (latent factor mixed model [LFMM], RDA and loci with high AFD). This resulted in a set of 10,245 SNPs and 3,245 genes. Here we also recalculated outliers from Ozerov et al. (2022) data using CSS approach. For subsequent analyses of the CSS pool‐seq dataset, we focused on genomic regions rather than individual outlier SNPs. A genomic region was considered potentially under selection if it encompassed ≥ 3 outlier SNPs, spaced less than 50 kb apart. This approach is expected to minimise the false‐positive identification of outlier SNPs.

### 2.7 Dynamic Outlier Slicing of Functional and GO Enrichment

To assess the impact of selecting different outlier thresholds on functional and GO enrichment outcomes, we developed a dynamic outlier slicing procedure. This entailed a comprehensive exploration of outlier cutoffs, using standard deviation values ranging from 2 to 6, with increments of 0.05 for the raw CSS statistics. This exploration resulted in the definition of a total of 81 distinct thresholds, thereby generating 81 different subsets of outlier SNPs. After obtaining the 81 groups of loci, we subsequently evaluated the overrepresentation and underrepresentation of SNPs within specific annotation categories (Sequence Ontology Mappings for SNPeff as Regions and Effects). We conducted chi‐squared tests, comparing each subset of SNPs against the complete SNP dataset for each annotation category.

Similar to SNP annotation, we conducted GO enrichment analysis for each subset of outlier SNPs using clusterProfiler v.4.8.2 (Wu et al. 2021) package, leveraging the human gene database from org.Hs.eg.db package (Carlson et al. 2019). This analysis covered the three ontologies: MM, and biological process and a significance threshold of 0.05 (Benjamini‐Hochberg adjusted *p*‐value) was applied to determine enriched categories.

The dynamic outlier slicing, examination of the overrepresentation/underrepresentation of SNPs within variant annotation categories, and GO analyses for each subset of SNPs at each threshold was conducted for both the pool‐seq and the ind‐seq datasets. These analyses were executed using a specialised custom R script developed for this purpose (Supporting Information). The visualisation was executed through heatmaps with the function geom_tile() implemented in ggplot2 package (Wickham 2009), employing the tables that encompassed distinct categories for both the functional annotation of the variants and the GO terms across all SNP subsets.

## 3 Results

### 3.1 Allele Frequency Estimates From Individual Versus Pooled WGS

Altogether, our analyses comprised a total of 50 populations (42 pooled and 32 individual datasets; 24 lakes shared) distributed throughout Northern Europe (Figure 1A). The selection of the lakes for this study was based on pronounced contrasts in water colour and dissolved organic carbon (DOC) content (mean clear water lakes = 8.39; mean humic lakes = 29.65). DOC and water colour exhibited a very strong positive correlation, with a Pearson correlation coefficient of 0.96 (*p* < 2.2^−16^; Figure 1C).

The assessment of allele frequency correlation between the full ind‐seq and pool‐seq datasets for clear and humic lake populations separately, revealed very strong correlations between the ind‐seq and pool‐seq datasets. The Pearson correlation coefficients for ind‐seq and pool‐seq datasets was 0.92 (*p* < 2.2^−16^) and 0.93 (*p* < 2.2^−16^) for clear and humic lakes populations, respectively (Figure 1D). However, the correlation for the estimated allele frequency differences (|AFD|) between humic and clear‐water lakes for both datasets was weaker (*r* = 0.27), although highly significant (*p* < 2.2^−16^).

### 3.2 Genetic Diversity, Differentiation and Population Structure

**FIGURE 2:**
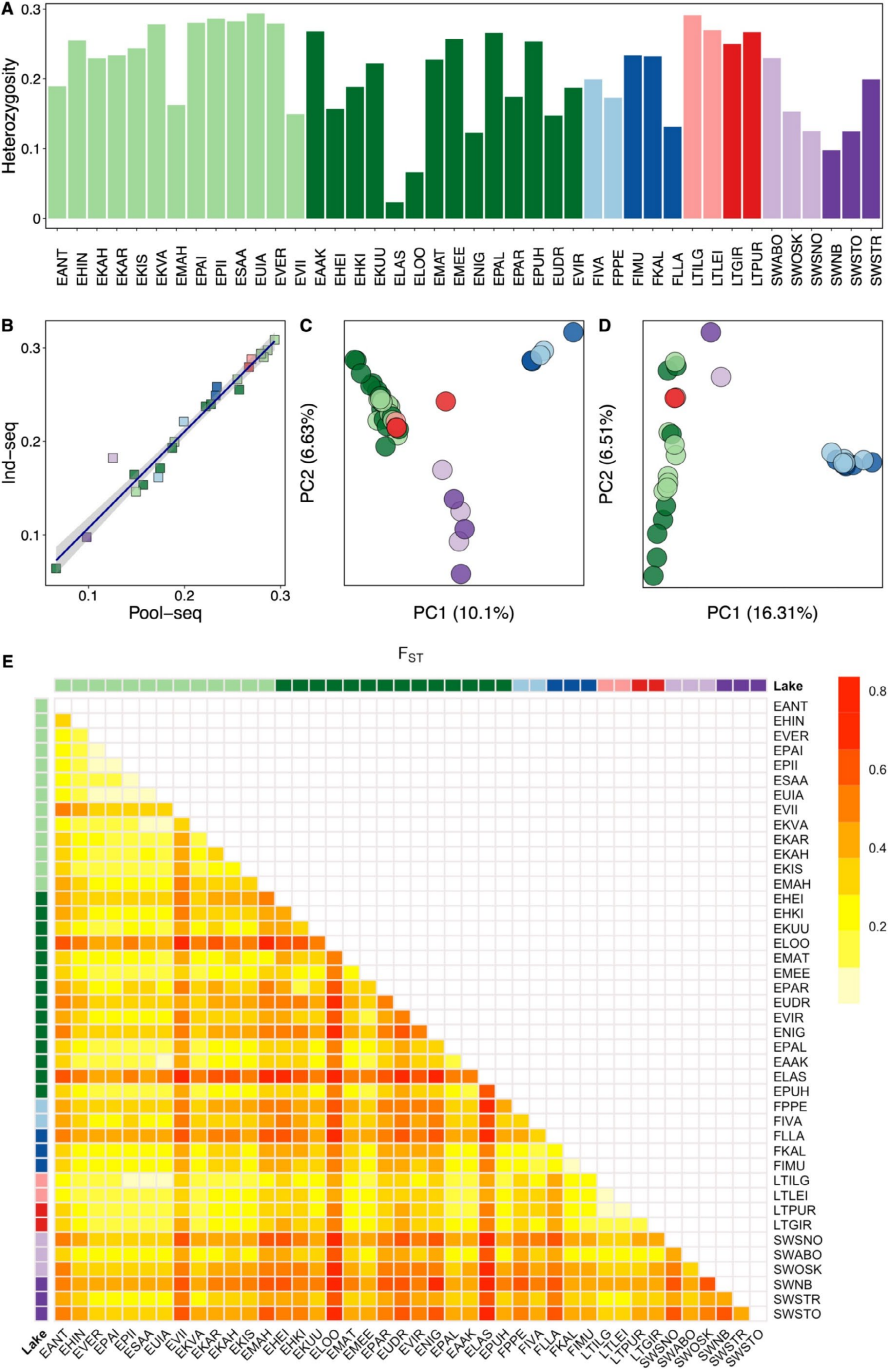
Genetic diversity and population differentiation. (A) Heterozygosity levels in populations analysed with pool‐seq. (B) Correlation of heterozygosity levels estimated for pool‐seq and ind‐seq datasets across the 24 common populations. Each data point represents a single population. (C) Principal component analysis (PCA) for populations studied using pool‐seq. (D) PCA for populations studied using ind‐seq. (E) Heatmap of *F*_ST_ values across all pairs of populations analysed with pool‐seq.

The evaluation of genetic diversity in the pool‐seq dataset revealed higher levels of genetic diversity in clear‐water lake populations than in humic lake populations (Table S2). For example, the mean estimated heterozygosity for clear‐water lakes was 0.23 ± 0.05, in contrast to 0.19 ± 0.07 in humic lakes (Wilcoxon test, *p* = 0.02) (Figure 2A). Similarly, π was higher in the clear‐water lakes compared to humic lakes (0.00139 ± 0.00017 vs. 0.00125 ± 0.00022, Wilcoxon test, *p* = 0.03). The Watterson's θ, and the number of segregating sites (S) were also higher for clear‐water lakes, but these differences were not statistically significant. To further compare diversity estimates between ind‐seq and pool‐seq datasets, we focused on the 24 shared populations. The correlation of estimated heterozygosities between pool‐seq and ind‐seq datasets was almost perfect (*r* = 0.98, *p* < 2.2^−16^, Figure 2B), indicating that WGS of a single individual per lake provided essentially the same information on population diversity as pool‐seq.

Analyses of the 42 populations using pool‐seq dataset revealed a substantial level of genetic differentiation, with half of the pair‐wise comparisons exhibiting average *F*_ST_ values higher than 0.35 (Figure 2E, Table S3). This pattern likely reflects the impact of strong genetic drift, combined with the limited connectivity and gene flow between the lakes. Furthermore, the average *F*_ST_ between countries ranged from 0.257 (Estonia‐Lithuania) to 0.454 (Sweden‐Finland). Within each country, mean *F*_ST_ values varied between 0.003 (Lithuania: LTLEI‐LTPUR) and 0.860 (Estonia: ELOO‐ELAS).

The PC analysis based on the allele frequency estimates of the 42 populations using pool‐seq dataset revealed three main clusters irrespective of the humic content of the lake. PC 1 and 2 accounted for 10.1% and 6.63% of variation of pool‐seq data, respectively (Figure 2C). The populations from Estonia and Lithuania clustered together, except of one lake (Girutiškis: LTGIR), which appeared to be further away from the main cluster. Additionally, populations from Finland and Sweden formed each separate clusters. Similar patterns were also observed for the ind‐seq dataset, where samples from each country clustered together. In this case, PC1 and PC2 explained 16.31% and 6.51% of the variance, respectively (Figure 2D).

### 3.3 The Frequency and Chromosomal Distribution of Composite Selection Signatures: Pool‐Seq Versus. Ind‐Seq

**FIGURE 3:**
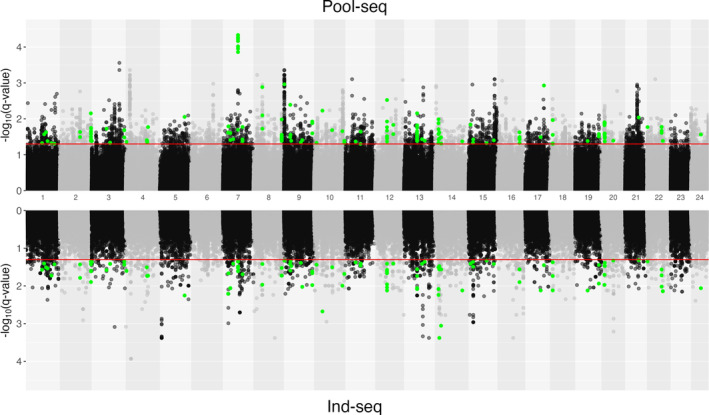
Mirrored Manhattan plots of composite selection signatures (CSS). The upper panel presents a Manhattan plot of *q*‐values for the pool‐seq dataset, while the lower panel shows a Manhattan plot of *q*‐values for the ind‐seq dataset. The genome‐wide significance level is set at ‐log_10_(0.05) = 1.3, plotted as the red line. Significant SNPs common to both datasets are highlighted in green.

The identification of outlier loci by combining AFD and RDA analyses using CSS, revealed 3,556 and 2,040 outlier SNPs for pool‐seq and ind‐seq datasets (*q*‐ < 0.05), respectively, distributed across all 24 chromosomes, with no SNPs identified in unplaced scaffolds (Figure 3; Tables S4 and S5). However, only 160 SNPs were found to be common between the two datasets (Table S6). In contrast, a total of 993 and 619 genes containing outlier SNPs were identified in the pool‐seq and ind‐seq datasets, respectively, with 148 common genes (Table S7). In the pool‐seq dataset, the frequency of outliers was not uniform among chromosomes, with 11 and five chromosomes displayed a significant deficiency and excess, respectively (deficiency: CHRs 1, 5, 6, 12, 13, 14, 16, 17, 18, 19 and 23; excess: CHRs 9, 10, 15, 21 and 24). Meanwhile, for ind‐seq, seven chromosomes showed significant deficiency (CHRs 2, 3, 6, 11, 17, 18 and 21), and five chromosomes displayed a significant excess of candidate SNPs (CHRs 7, 10, 13, 14, 20). The visual inspection of Manhattan plots revealed a pronounced clustering of highly significant outliers within the pool‐seq dataset compared to the ind‐seq dataset. This pattern is likely due to the larger number of studied populations, which increases the power to detect outliers, along with more accurate allele frequency estimation (see Table S8 for Supporting Information).

The CSS analysis conducted on pool‐seq dataset confirmed the outlier status of a small number of SNPs and genes previously linked to the adaptation differences in humic/clear‐water lakes for the Eurasian perch (Ozerov et al. 2022). In total, 493 outlier SNPs and 447 outlier genes were found to overlap between CSS pool‐seq and the results reported by Ozerov et al. (2022), while 1769 putative outlier SNPs and 580 outlier genes overlapped between CSS ind‐seq and Ozerov et al. (2022) dataset (Tables S9 and S10).

Subsequently, upon identifying candidate genomic regions potentially under selection, we investigated the patterns of genetic diversity within them to ascertain whether the reduction in genetic diversity was more pronounced in humic or clear‐water environments, aiming to identify the specific habitat where adaptation most likely occurred. This analysis revealed a total of 324 candidate regions under selection, with 277 displaying reduced heterozygosity levels in humic environment, while 47 regions exhibited lower heterozygosity in clear‐water habitat (Fisher's exact test for equal proportion, *p* = 0.0001). The size of the genomic regions identified ranged from < 100 bp to 462.4 kb (median = 18.4 kb; mean = 31.9 kb). The number of outlier SNPs within these regions ranged from 3 to 143 (median = 5; mean = 8.3, *r* = 0.72, *p* < 2.2^−16^, Figure S3). The distribution of genes within these regions was as follows: 74 regions contained no genes, 142 regions contained a single gene, 55 regions contained two genes, 29 regions contained three genes and 24 regions harboured more than four genes (Table S11).

### 3.4 Functional and GO Enrichment Analysis Using Dynamic Outlier Slicing

**FIGURE 4:**
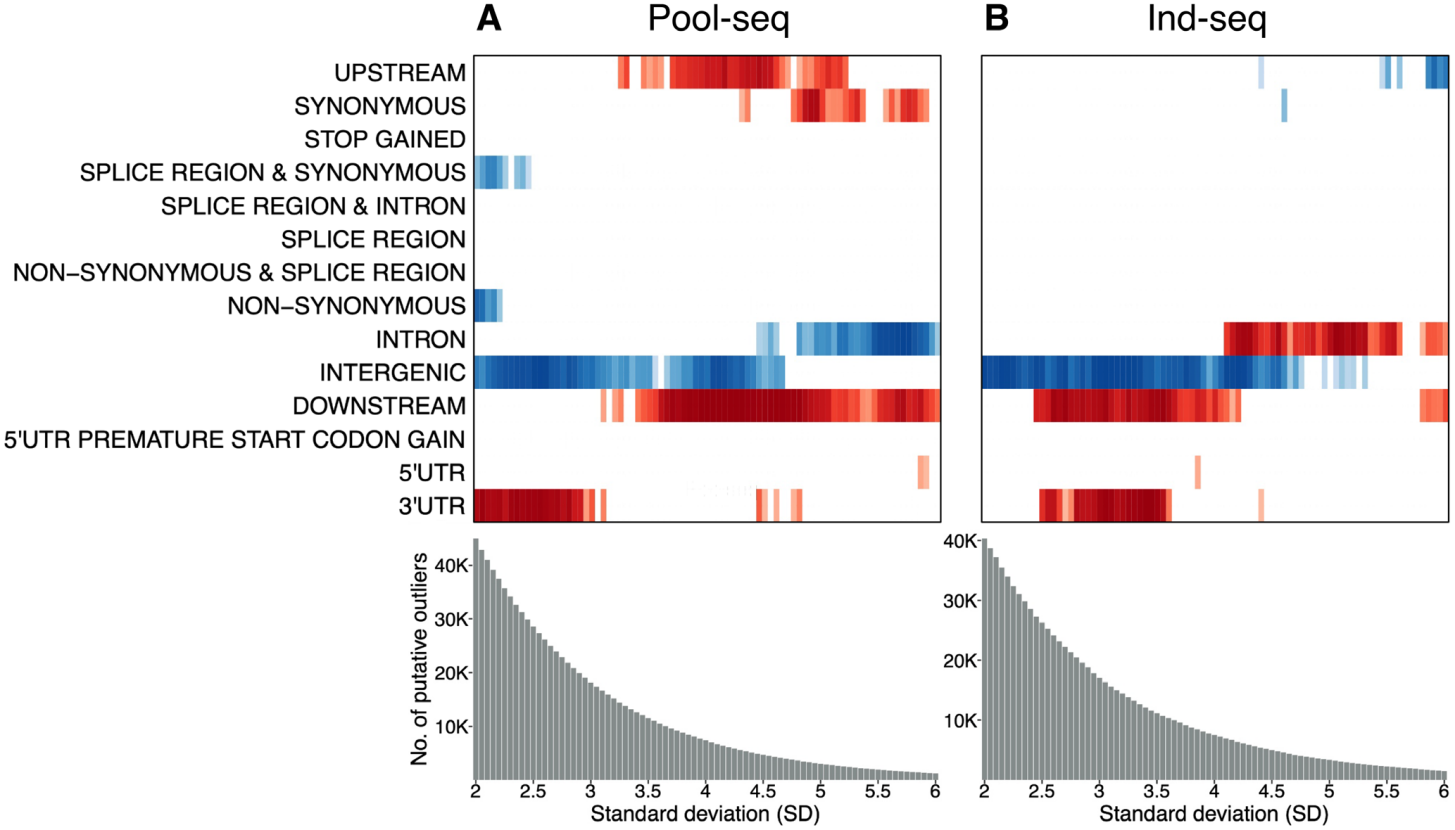
Dynamic outlier slicing for genome annotation. Overrepresentation and underrepresentation of candidate SNPs across 81 distinct cutoffs for the raw CSS statistics are shown for (A) pool‐seq and (B) ind‐seq, respectively. These thresholds were determined using standard deviation values ranging from 2 to 6, with increments of 0.05. The analysis covers 14 specific annotation categories (Sequence Ontology Mappings for SNPeff as Regions and Effects) for both pool‐seq and ind‐seq datasets. The colour red represents enrichment, while the colour blue indicates depletion of annotation categories.

Next, we explored the effect of outlier calling stringency on functional and GO enrichment analysis. In the pool‐seq data, five SNP annotation categories (upstream, downstream, synonymous, 5′UTR, and 3′UTR) showed significant overrepresentation of outliers. 3′UTR was enriched at lower thresholds (SD 2–4.8), while upstream, synonymous and downstream regions at higher thresholds (SD 3.1–6) and 5′UTR only at SD 5.85 and 5.9. Conversely, splice region & synonymous, non‐synonymous, intergenic and intron were consistently depleted, with splice region & synonymous and non‐synonymous depleted at SD 2–2.45; intergenic regions at SD 2–4.65, and intron at SD 4.45–6 (Figure 4A).

In the ind‐seq dataset, four SNP annotation categories (downstream, 5′UTR, 3′UTR, and intron) showed significant enrichment. Downstream outliers were enriched at SD 2.45–6, while 3′UTR and 5′UTR were enriched in a narrower ranges (SD 2.5–4.4 and SD 3.85, respectively) than pool‐seq. Notably, intronic outliers showed enrichment across several thresholds (SD 4.1–6), contrasting with their depletion in pool‐seq. Depletion was observed in upstream regions (SD 4.4–6), synonymous category (SD 6) and intergenic regions (SD 2–5.3), similar to the pool‐seq pattern (Figure 4B).

**FIGURE 5:**
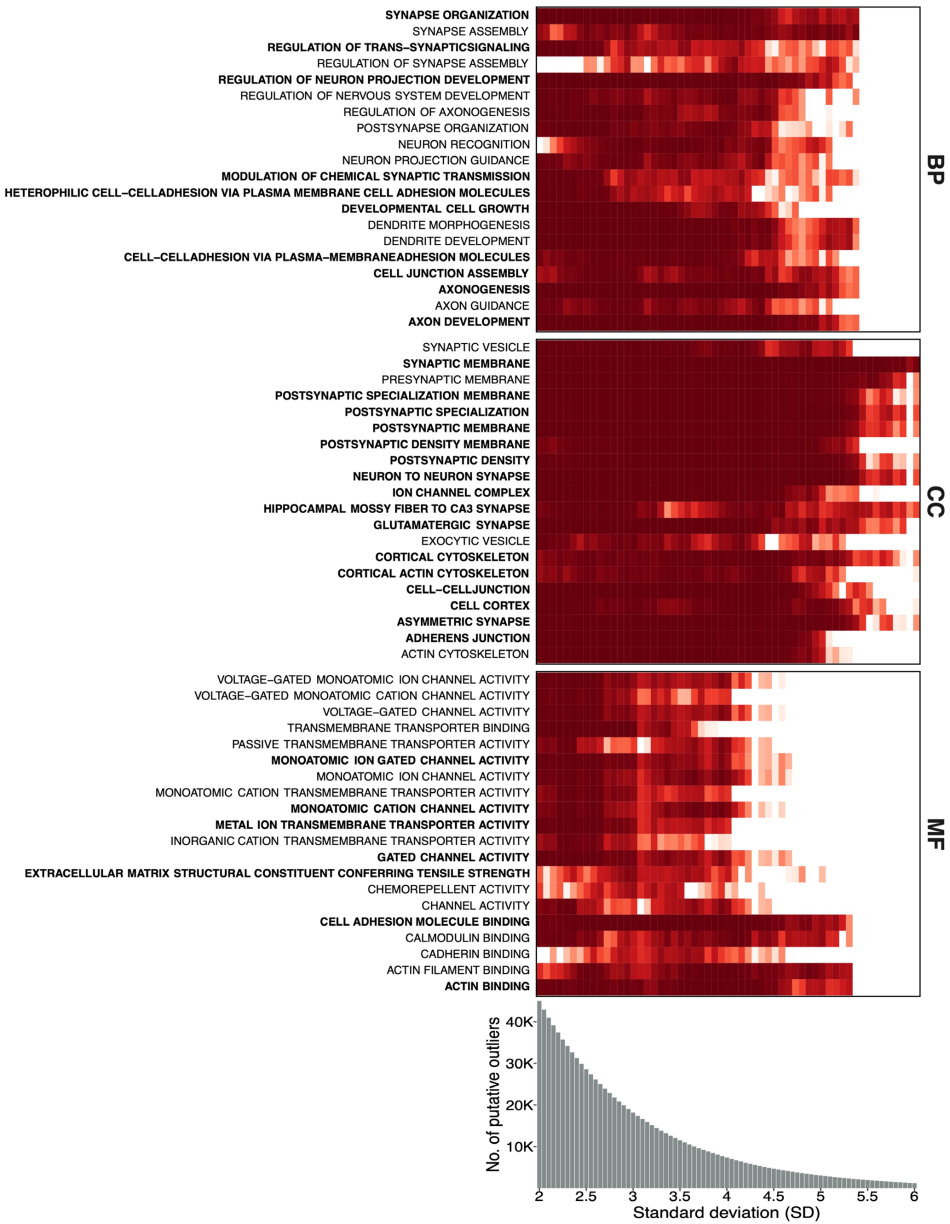
Dynamic outlier slicing for Gene Ontology (GO) enrichment analysis in the pool‐seq dataset. The 20 most prevalent GO terms are plotted across the three ontologies: Molecular function (MM), cellular component (CC) and biological process (BP) for the pool‐seq dataset. GO terms in bold represents categories common to both pool‐seq and ind‐seq datasets.

The application of dynamic outlier slicing for GO enrichment analysis within pool‐seq dataset revealed enrichment of outliers for a large number of GO terms. Specifically, the BP category revealed enrichment of multiple terms (*n* = 328) from threshold SD 2–4.35, with several of its most significant terms associated with the nervous system, such as synapse organisation, regulation of nervous system development, dendrite development and morphogenesis, synapse assembly, and axon development, among others. The CC category consisted the GO terms (*n* = 115) exhibiting enrichment to the highest thresholds (SD up to 4.8), featuring with synaptic processes, such as synaptic membrane, presynaptic membrane, postsynaptic density, and hippocampal mossy fibre to CA3 synapse. MF category exhibited enrichment of various terms (*n* = 73) up to SD 4.3 including cell adhesion molecule binding, calmodulin binding, gated channel activity, transmembrane transporter activity, voltage‐gated channel activity, ion channel activity, actin filament binding and actin binding (Table S12, Figure 5).

On the other hand, when employing dynamic outlier slicing for GO enrichment within ind‐seq dataset, we observed that BP (*n* = 304) and CC (*n* = 130) ontologies were enriched to more stringent levels compared to pool‐seq (Table S13, Figure S2). For instance, the BP category exhibited enrichment of multiple terms ranging from threshold SD 2–5.25, while the CC category showed enrichment from SD 2 up to 6. Meanwhile, the MF category (*n* = 92) displayed enrichment up to a threshold of 3.2. Furthermore, a majority of the shared terms between pool‐seq and ind‐seq were observed in CC with 16 common terms, followed by BP with 10 terms and MF with 7 terms. Consistent with pool‐seq results, BP showed several categories associated with the nervous system, while CC terms were related to synapsis function. MF exhibited enrichment in terms such as protein kinase activity, semaphorin receptor activity and monoatomic cation channel activity, among others.

### 3.5 Genes Previously Implicated in Adaptation to Humic Environment

**FIGURE 6:**
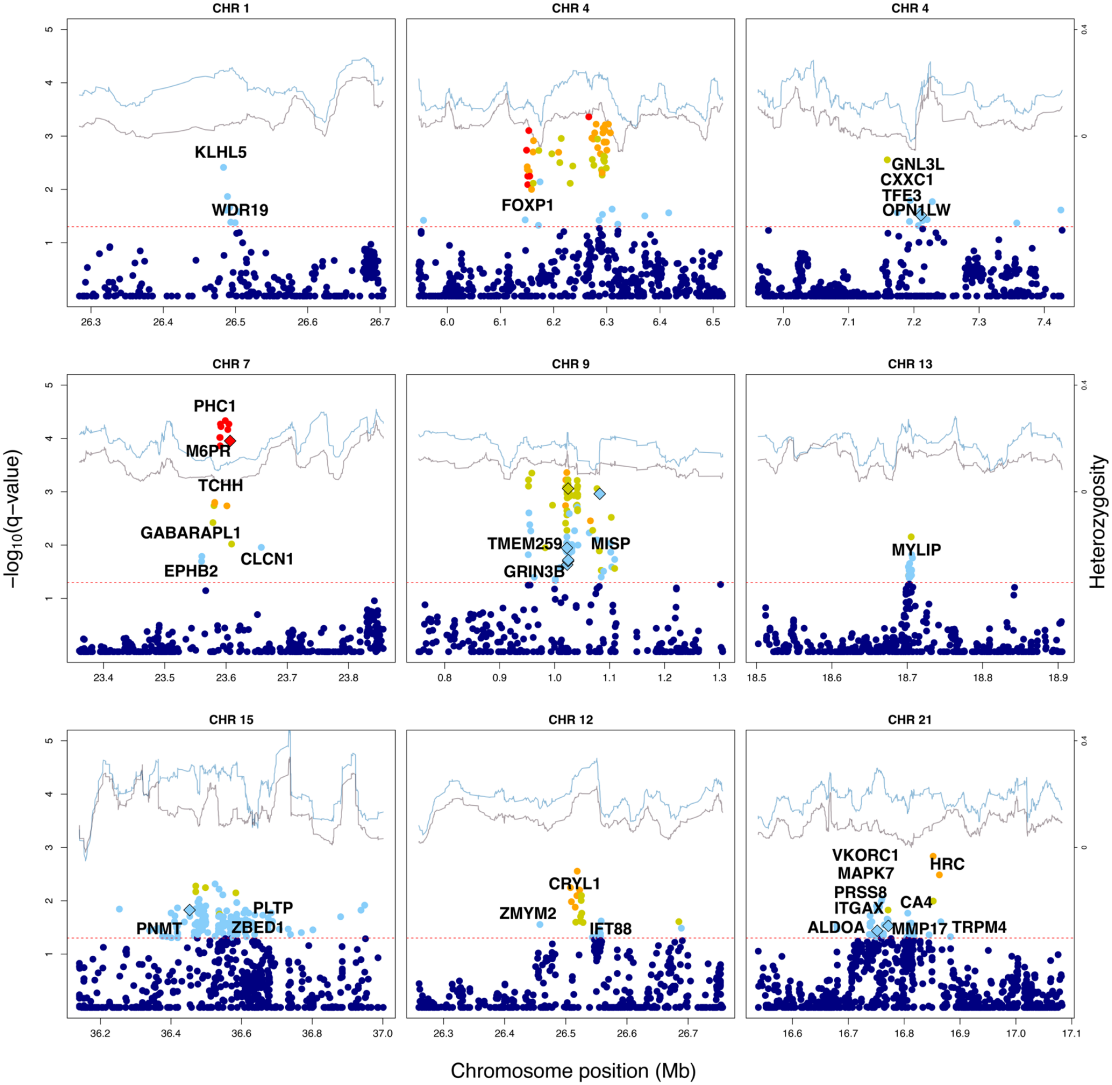
Detailed visualisation of significant genomic regions in the CSS analysis for pool‐seq. The upper portion of each plot features lines representing heterozygosity levels, calculated using windows of 25 SNPs, displayed on the secondary *y*‐axis for populations from clear (light blue line) and humic (dark grey line) environments. The genome‐wide significance threshold is set at ‐log_10_(0.05) = 1.3, represented by a dashed red line. Significant SNPs are colour‐coded to represent allele frequency differences (AFD) between the populations: Red for AFD > 0.4, orange for AFD ≤ 0.35, yellow for AFD ≥ 0.3, and light blue for AFD < 0.3. Missense mutations are shown as filled diamond shapes.

Despite the relatively moderate overlap of outlier SNPs between CSS pool‐seq and the study by Ozerov et al. (2022) several candidate regions were identified in both analyses. For instance, on chromosome 1, we localised a 21 kb region exhibited reduced genetic diversity in humic populations (Figure 6A), harbouring the gene *WDR19*. Mutations in *WDR19* have been linked to retinal diseases that can lead to blindness in humans (Coussa et al. 2013; Sajovic et al. 2023) and associated to adaptation for nocturnal vision of night herons (Luo et al. 2022). Similarly, candidate regions containing *CDON* (CHR. 2; −log_10_ = 1.99) and *CHD7* (CHR. 11; −log_10_ = 2.12) were also linked to nocturnal vision adaptation in herons (Luo et al. 2022) and identified in Ozerov et al. (2022).

Another region, covering 174 kb and encompassing 55 outlier SNPs, was identified on chromosome 4 (Figure 6B). This region showed a notable reduction in genetic diversity within humic lake populations and harboured the *FOXP1* gene. *FOXP1* is a member of the highly conserved FOX gene family, which play pivotal roles in several developmental and homeostatic processes (Hannenhalli and Kaestner 2009). Specifically, the *FOXP1* gene has shown to be differentially expressed in songbird and human brain regions crucial for the developmental involved in speech and language (Horn et al. 2010). Furthermore, *FOXP* genes exert regulatory influence over the adaptative immune system (Pignata and Romano 2014). On the same chromosome, within a region of 69, 492 kb apart from the previous region, we identified the genes *CXXC1*, *TFE3*, *GNL3L* and *OPN1LW*, the latter is one of the four red‐sensitive opsin‐like gene orthologues, also identified by Ozerov et al. (2022) (Figure 6C).

One of the most significant regions in pool‐seq dataset was localised in chromosome 7 (−log_10_ = 4.33), within a 97.6 kb segment harbouring 18 outlier SNPs. This region included the *TCHH*, *PHC1* and *M6PR* genes and showed reduced genetic diversity in humic populations (Figure 6D). The *TCHH* gene is known for its role in human hair and skin development (Medland et al. 2009; Wu et al. 2016). *PHC1* is a member of the Polycomb group (PcG) of proteins, which act antagonistically to Trithorax group (TrxG) components in gene regulation during cell differentiation and development (Schuettengruber et al. 2017); while *M6PR* belongs to P‐type lectins, which plays crucial role in lysosomal enzyme transport, bacterial resistance and viral entry (Liu et al. 2023). Additionally, a 157 kb region on chromosome 9 containing 90 outlier SNPs harbours the genes *MISP*, *GRIN3B* and *TMEM259* (Figure 6E). *GRIN3B* gene, implicated in synaptic development and neurotransmission (Chikina, Robinson, and Clark 2016), *TMEM259* is involved in inhibiting neuron death and positive regulation of ERAD pathway (Zhu et al. 2017).

We also identified *MYLIP* on chromosome 13 (−log_10_ = 2.15) within a region of 5.4 kb and 14 outlier SNPs (Figure 6F). This region exhibited one of the highest allele frequency differences between humic and clear‐water perch in the 32 genome dataset (Ozerov et al. 2022). The *MYLIP* gene, also known as *IDOL*, plays role in lipid metabolism (Lindholm, Bornhauser, and Korhonen 2009; van Loon, Lindholm, and Zelcer 2019) and has been implicated in the early embryonic development of zebrafish, particularly in calcium‐dependent mechanisms during gastrulation (Knowlton, Chan, and Kelly 2003).

### 3.6 Newly Identified Genes Potentially Contributing to Humic Adaptation

The CSS analysis conducted on the pool‐seq dataset revealed 3,063 novel outlier SNPs and 546 genes that had not been previously detected in Eurasian perch. On chromosome 12, a 100 kb with 24 outlier SNPs displayed lower genetic diversity in humic populations. This region contains the *IFT88*, *ZMYM2* and *CRYL1* genes (Figure 6H). The *IFT88* is involved on transport of opsin molecules in zebrafish (Sukumaran and Perkins 2009; Hudak et al. 2010) and has shown evidence of positive selection in secondary adaptation to temperate environments in non‐Antarctic icefish, following specialisation to Antarctic conditions, including unique polar light–dark regime (Rivera‐Colón et al. 2023). Additionally, the *CRYL1* gene, initially discovered in rabbit and hare lenses, encodes the Lambda‐crystallin protein, related to hydroxyacyl‐coenzyme A dehydrogenases (Mulders et al. 1988). *CRYL1* has shown increased gene expression in retina cells following exposure to cadmium in zebrafish (Scudiero et al. 2017). On chromosome 15, we identified a significant cluster of 147 SNPs within a 662 kb region, which exhibited reduced genetic diversity in humic population throughout most of the area (Figure 6G). This region contains the *PNMT* gene, associated with predatory feeding behaviour and aggression in mandarin fishes (He et al. 2020). Additionally, the region encompasses the *PLTP* gene, which is involved in lipid and lipoprotein metabolism (Vuletic et al. 2003; Albers, Vuletic, and Cheung 2012) and influences cognitive performance in humans (Tong et al. 2015).

Other regions exhibiting significant differences in genetic diversity include a 145 kb region on chromosome 21, with 59 outlier SNPs containing 10 genes: *ITGAX*, *ALDOA*, *VKORC1*, *MAPK7*, *PRSS8*, *PAX6*, *MMP17*, *CA4*, *HRC*, *TRPM4* (Figure 6I). Among these, *ALDOA* and *MAPK7* have been involved in the response to hypoxic environments in Tibetan fish species, common carp and Schizothoracines fishes (Zhang et al. 2017; Hung et al. 2022; Martínez Sosa and Pilot 2023). The *TRPM4* gene has been shown to be associated with response to light stimuli and inflammation in teleosts (You et al. 2020; Li et al. 2021), and is a candidate for putative thermosensors with evidence of diversifying selection in Antarctic fishes (Cryonotothenioidea) (York and Zakon 2022). *PAX6* has transcriptional control over crystallin genes in jellyfish, and is involved in vertebrate lens morphogenesis (Cvekl et al. 2017; Hahn et al. 2017).

### 3.7 Newly Identified Genes Potentially Involved in the Clear‐Water Adaptation

Among the regions that showed lower genetic diversity in clear water environment, some noteworthy candidates were identified. On chromosome 1, a 10 kb region contained 3 outliers SNPs and the *SART1* gene, which is involved in cell death and vision‐related pathways in zebrafish (Henson and Taylor 2020). Chromosome 2 featured a 74 kb region with 26 outliers SNPs harbouring the *GRIK4* gene, associated with ion regulation (Ding et al. 2023). On chromosome 12, a 5 kb region included the *GPR183* gene, also known as the Epstein–Barr virus‐induced G‐protein coupled receptor 2, which plays a role in immune response regulation (Chen, Huang, and Li 2022). Chromosome 22 had 26 kb region containing the *NCOA2* gene, known to modulate lipid metabolism and control energy homeostasis in pigs, thus playing a significant role in the regulation of metabolic processes (Ramayo‐Caldas et al. 2014). Finally, on chromosome 24, a 25 kb region with 76 outlier SNPs harboured the *FLO11* gene, which is implicated in cell–cell and cell‐surface adhesion (Halme et al. 2004).

## 4 Discussion

This study focused on detecting novel and confirming known genomic signatures of selection linked to humic substances in Eurasian perch, leveraging a large set of populations living in humic and clear‐water environments. Specifically, we aimed to evaluate a newly developed outlier slicing approach by testing how different outlier thresholds influence the outcomes of functional enrichment, and to compare the efficacy of pooled versus individual sequencing methodologies for outlier detection. Our analysis revealed regional population structure and genetic isolation among most populations, consistent with strong genetic drift, combined with limited connectivity between the lakes. Nevertheless, despite substantial drift, analyses of a large number of populations enabled the identification of consistent signals of selection involving numerous genomic regions associated with humic adaptation.

One of the primary challenges in population genetics is differentiating between genomic regions influenced by selection and those representing neutral genetic variation (Beaumont 2005; Oleksyk, Smith, and O'Brien 2010; Ahrens et al. 2018; Weigand and Leese 2018). Various factors can significantly impact the effectiveness of identifying true footprints of selection, such as variation in recombination, mutation rate, gene flow, linkage disequilibrium and demographic history (Hoban et al. 2016). Furthermore, pronounced genetic structure and differentiation, as well as small population size where drift can have strong effects, may obscure signals of selection in the genome. Following a similar approach as Ozerov et al. (2022), we selected geographically neighbouring lakes with drastic differences in humic content. This design allowed us to compare fish living in markedly different environments, while accounting for comparable phylogenetic backgrounds. To mitigate the effect of drift, we incorporated data from 42 populations spanning various locations across four countries. This extensive population sampling is expected to significantly improve the power of both allele frequency‐based outlier tests and GEA approaches (De Mita et al. 2013; Whitlock and Lotterhos 2015). Additionally, by encompassing diverse populations, we aimed to capture a wider spectrum of adaptive variation, providing deeper insights into the adaptive potential of the species (Lotterhos and Whitlock 2014). While we did not focus on population‐specific adaptations due to the different challenges they present, our study system provides a solid foundation for future investigations into both common and regional adaptive signals.

### 4.1 Genetic Evidence for Adaptation to Humic and Clear Water Environments

We combined signals from allele frequency‐based outlier tests with GEA approaches and identified 3,556 SNPs outliers, of which 2,679 were distributed across 324 genomic regions involving 468 genes suggesting that adaptation to humic environment likely involves large number of regions scattered across the genome. This is consistent with the earlier analyses of selective sweeps associated with humic environment involving 32 individual perch genomes (Ozerov et al. 2022) as 447 common genes were identified by both studies as potential targets influenced by divergent selection. Yet, by directly comparing the signatures of selection inferred from individual genomes (Ozerov et al. 2022) and whole‐genome pool‐seq data, the latter approach revealed more distinct peaks of selection across several genomic regions (Figure 3). This is likely caused by more accurate representation of AF involving higher number of populations in pool‐seq data (42 vs. 32 populations for pool‐seq and ind‐seq, respectively). Among the identified regions, 277 displayed reduced heterozygosity levels in humic populations, while 47 regions exhibited lower heterozygosity in clear water populations (Fisher's exact probability test, *p* = 0.0001). Thus, an excess of reduced genetic variation in humic environment within candidate regions supports the hypothesis that adaptation to extreme humic environment represents a prevalent type of selection in the studied system. However, a smaller proportion of adaptation signals were also associated with clear‐water environment. Clear‐water environment are complex habitats where many biotic factors, such as competition with other fish species, mating preferences, rich parasite and predator community, may act as important selective agents (Magnhagen and Heibo 2004; Horppila et al. 2011; Ranåker et al. 2012, 2014). For example, earlier studies have shown that perch populations in clear‐water lakes often host high prevalence of Diplostomid eye parasites in contrast to humic lakes that typically lack eye flukes (Noreikiene et al. 2020; Diaz‐Suarez et al. 2024). Furthermore, evaluation of the genetic diversity within candidate regions revealed many areas with rather similar heterozygosity estimates in humic and clear‐water lakes. Thus, these regions may lack a typical characteristic of hard sweep and may be more compatible with more complex history of selection, for example soft sweeps where the local reduction of diversity is not prominent (Hermisson and Pennings 2005). In terms of geological history, clear‐water habitats likely represent the ancestral habitat type, as the entire study area was covered by an ice sheet 12,000–14,000 years ago (Stroeven et al. 2016). Following the ice melt, the initial aquatic habitats in this region were predominantly consisted of cold, oligotrophic, clear‐water lakes.

### 4.2 Dynamic Outlier Slicing of Functional Annotation Categories

Genetic variants differ in their likelihood of influencing phenotypic effects and being targets of selection (Nielsen 2005). To better understand the non‐random distribution of outliers and the robustness of the enrichment signals, we systematically evaluated the enrichment spanning multiple outlier thresholds. We observed a consistent overrepresentation of outliers within the upstream, downstream and 3′UTR regions across various outlier thresholds. This finding suggests that our outlier identification strategy combining different approaches was robust across various significance thresholds since random set of SNPs are not expected to generate significant enrichments. Furthermore, these results strongly indicate that regulatory elements play an important role in perch facilitating adaptation to humic substances. Interestingly, similar patterns were observed when dynamic outlier slicing was applied to an earlier dataset of Ozerov et al. (2022), which consisted of 32 individual perch genomes. In addition, we identified significant enrichment of outliers for synonymous variants, suggesting its relevance in adaptive processes. This finding aligns with recent evidence suggesting that synonymous mutations may also have functional significance (Bailey, Alonso Morales, and Kassen 2021; Shen et al. 2022), despite being frequently regarded as neutral (Williamson et al. 2005; Bailey, Alonso Morales, and Kassen 2021). Indeed, many earlier studies indicate that silent sites are not completely neutral and are shaped by purifying evolution (Eőry, Halligan, and Keightley 2010; Pollard et al. 2010; Künstner, Nabholz, and Ellegren 2011; Lawrie et al. 2013; Dutoit et al. 2017). Finally, the underrepresentation of outliers across intergenic and intronic regions aligns well with available knowledge since these regions have shown to mostly evolve neutrally, and being less frequently targets of selection (Andolfatto 2005; Haddrill, Bachtrog, and Andolfatto 2008).

### 4.3 Dynamic Outlier Slicing of GO Categories

Dynamic outlier slicing of GO categories revealed significant enrichment of large number of GO terms. Specifically, several terms associated with nervous system functions in the BP category. The nervous system plays a pivotal role in animal adaptation and evolution, controlling sensory, motor, and cognitive functions within the brain and spinal cord. Extensive research supports the intricate connection between environmental conditions, sensory systems, speciation and adaptation in aquatic organisms (Boughman 2002; Borghezan et al. 2021). The abundance of terms associated with the nervous system in our study suggests the potential involvement of sensory modalities in adapting to the humic environment. Furthermore, CCs also included terms such as neuron‐to‐neuron synapse and synaptic membrane, supporting the notion of genes playing roles in sensory system. On the other hand, the presence of several terms associated with ion channel complexes within both CC and the MF categories (such as ion channel complex, monoatomic ion channel activity, monoatomic cation channel activity, calmodulin binding) suggests the involvement of gene families in osmoregulation and ion balance. Given the extreme differences in pH levels between humic (typically pH 4–5) and clear‐water lakes (typically pH 6–8), it was anticipated that gene families related to osmoregulation would exhibit signs of selection. Similar patterns were found by Ozerov et al. (2022 where some of the top 10 most significant GO terms included nervous system development (BP); actin and calmodulin binding (MF) and cell junction, including nervous tissues (CC).

### 4.4 Individual Candidate Genes

Similar to previous research, this study found that the signatures of selection related to humic substances are distributed throughout the entire genome, involving numerous genes with diverse functions. Although we found relatively small overlap of outlier SNPs between our CSS Pool‐seq analysis and the study by Ozerov et al. (2022), several candidate regions were identified in both studies, highlighting their potential role in adaptive processes to humic environments. For instance, genes such as *WDR19*, *CDON*, *CHD7* and *OPN1LW*, which are involved in visual traits, may be linked to adaptive changes in the extreme visual conditions of humic environments, akin to observations in other fish species inhabiting blackwater environments (Marques et al. 2017). Moreover, genes like *FOXP1*, *GRIN3B* and *TMEM259*, which are important for synaptic development and neuronal and synaptic survival, suggest roles in neurological adaptations. The *MYLIP* gene, shows high divergence between humic and clear‐water lake populations, consistent with findings from Ozerov et al. (2022), indicating its potential association with compensating for Ca^2+^ deficiency during embryonic development in humic lakes.

Among newly discovered candidates were genes involved in opsin molecule transport, such as *IFT88*, and those showing increased expression in zebrafish retina cells under cadmium exposure, such as *CRYL1*. *PAX6* is critical for crystallin gene transcription and lens morphogenesis in vertebrates, which also suggest that selective pressures influence changes in their visual sensitivity to better align with the ambient light conditions (Escobar‐Camacho et al. 2017). Additionally, genes responding to hypoxic conditions in fish, such as *ALDOA* and *MAPK7*, may be involved in the adaptation of fish to oxygen stratification in humic lakes (Kankaala et al. 2006). This is supported by the finding that changes in oxidative and physiological parameters, which are associated with the presence of humic substances, have been observed in other fish species. The findings highlight the intricate and multifaceted nature of genetic adaptation to challenging environmental conditions, emphasising the complexity and breadth of adaptive responses to humic environments.

### 4.5 Analysis of Individual Versus Pooled Genomes: Genetic Diversity and Differentiation

The accurate estimation of AF is a crucial starting point for deciphering evolutionary processes such as genetic drift, gene flow, and natural selection within natural populations (Nielsen and Slatkin 2013). Yet, whole‐genome analyses based on just a single individual per population have been able to reveal neutral and adaptive evolutionary processes at remarkable detail (Jones et al. 2012; Ozerov et al. 2022). In this study, a high correlation observed in AF was observed between the pool‐seq and ind‐seq datasets, indicating general consistency in AF across populations inhabiting clear‐water and humic lakes. However, the estimated allele frequency differences in pool‐seq and ind‐seq datasets showed a much weaker correlation, which also was reflected by limited overlap between identified outlier SNPs. The high genetic differentiation and structure observed among the studied populations are consistent with the results of a previous study by Ozerov et al. (2022), which used populations with the same geographical origin. This pattern of strong genetic differentiation provides compelling evidence for importance of genetic drift, combined with isolation and either limited or non‐existent gene flow among populations. More generally, the similarity in genetic structuring between the pool‐seq and ind‐seq results underscores the reliability of pool‐seq in capturing population genetic patterns shown also by other studies (Dorant et al. 2019; Chen, Parejo et al. 2022).

The analysis of genetic diversity based on pool‐seq datasets also provided important insights into the genetic diversity of populations inhabiting different habitats. Our findings revealed that perch living in clear water lakes exhibited on average higher levels of genetic diversity compared to their conspecifics in humic lakes. A similar trend was observed by Ozerov et al. (2022). The reduced genetic diversity observed in dark lakes is likely associated with the extreme humic environment where low pH may cause failures in perch recruitment (Rask 1984; Rask et al. 2014). Thus, the genomic data supports the notion of extreme environment of highly humic lakes for Eurasian perch. Furthermore, our analyses revealed almost perfect correlation of genetic diversity estimates between pool‐seq and ind‐seq data across 24 populations. This reflects the power of WGS for accurate estimation of genetic diversity and suggests the potential of using a very small number of individuals per population for future genetic monitoring.

## 5 Conclusions

We aimed to characterise the genetic footprints of selection associated with contrasting aquatic environments in Eurasian perch in order to enhance our understanding of the evolutionary dynamics of humic adaptation. The comparison between pool‐seq and ind‐seq showed high correlation in diversity and allele frequency, as well as congruency in population genetic structuring patterns. The dynamic outlier slicing approach revealed an overrepresentation of outliers in the regions of upstream, downstream, synonymous, 5′UTR and 3′UTR, while four annotation categories (splice region & synonymous, non‐synonymous, intergenic and intron) were underrepresented among outliers. Additionally, the GO analysis indicated several enriched terms for BP and CC related to nervous system and synaptic processes. The MF category included gated channel activity, transmembrane transporter activity, voltage‐gated channel activity, ion channel activity, among others. Several genes identified in this study were consistent with previous work. Overall, these findings highlight the complex and diverse nature of genetic adaptation to challenging humic environmental conditions.

## Author Contributions

Anti Vasemägi and Riho Gross devised and planned the study. María‐Eugenia López and Mikhail Ozerov conducted the bioinformatics and population genomic analyses. María‐Eugenia López and Anti Vasemägi collaborated in writing the initial draft of the manuscript. Lilian Pukk and Kristina Noreikiene provided essential assistance with sampling and laboratory work, contributing to the study design. All authors participated in revising and refining the manuscript.

## Conflicts of Interest

The authors declare no conflicts of interest.

## Supporting information


Figures S1‐S5.

Tables S1‐S13.

## Data Availability

Table S8 is available in Figshare repository at https://figshare.com/s/4a0ec54481b0b07831e3. The pool‐seq raw data are available at NCBI under the project ID PRJNA1019892.
