# Supplementary material for: Dynamic Outlier Slicing Allows Broader Exploration of Adaptive Divergence: A Comparison of Individual Genome and Pool‐Seq Data Linked to Humic Adaptation in Perch
Source: Mol Ecol. 2025 Jan 23;34(4):e17659. doi: 10.1111/mec.17659 (PMC11815547; doi:10.1111/mec.17659)
Supplement: Supplementary file 1 — Figures S1‐S5. [file MEC-34-e17659-s001.pdf]

MOLECULAR ECOLOGY

Supplemental Information for:

Dynamic outlier slicing allows broader exploration of adaptive divergence: a comparison of individual genome and pool-seq data linked to humic adaptation in perch

López María-Eugenia, Ozerov Mikhail, Pukk Lilian, Noreikiene Kristina, Gross Riho, Vasemägi Anti

Table of Contents:

**Supplementary Analyses and Methods**	
Assessment of the Impact of Pool Size on Genetic Diversity Estimates	Page 2
Chromosomal Distribution of Outlier SNPs	Page 2
Estimation of Correlations	Page 2
Fisher's Exact Test for Differences in Selection Signatures Between Humic and Clear-Water Environments	Page 3
Figure S1	Page 4
Figure S2	Page 5
Figure S3	Page 6
Figure S4	Page 6
Figure S5	Page 7
**Supplementary Scripts**	
R Script for Generating Files of SNPs Passing Each Threshold	Page 8 – 9
R Script for Calculating Enrichment and Deple- tion Across Different Thresholds.	Page 10 – 13

MOLECULAR ECOLOGY

Supplementary Analyses and Methods

Assessment of the Impact of Pool Size on Genetic Diversity Estimates

Three pools were constructed with sample sizes of ***n* = 10**, ***n* = 20**, and ***n* = 40** using samples from the Estonian EUDR population (Lake Udriku Suurjärv). Estimated heterozygosity values varied slightly across pools: $EUDR_{10}$ =0.1494; $EUDR_{20}$ = 0.1700 and $EUDR_{40}$ =0.1547. Pairwise F_{ST} values between all three possible pair combination gives were negative values, indicating minimal or no detectable genetic differentiation: $EUDR_{10}$/$EUDR_{20}$= -0.026, $EUDR_{10}$/$EUDR_{40}$= -0.029, and $EUDR_{20}$/$EUDR_{40}$= -0.008.

Chromosomal Distribution of Outlier SNPs

To evaluate the uniformity of outlier distribution across chromosomes, we conducted a Chi-square test using the *chisq.test* function in R. This analysis compared the observed number of outlier SNPs per chromosome to the expected counts, which were calculated based on the proportion of total SNPs on each chromosome relative to the genome-wide SNP count. The null hypothesis assumed that outliers were uniformly distributed across chromosomes. A significant Chi-square result indicates non-uniformity in the distribution of outlier SNPs.

Estimation of Correlations

We calculated Pearson's correlation coefficients to evaluate relationships among several variables, using the *cor.test* function in R. These variables included: (*1*) dissolved organic carbon (DOC) and water color; (*2*) allele frequency correlations between the full ind-seq and pool-seq datasets; (*3*) allele frequency differences (|AFD|) between the full ind-seq and pool-seq datasets; and (*4*) heterozygosities across the 24 overlapping population shared between the pool-seq and ind-seq datasets.

MOLECULAR ECOLOGY

Fisher's Exact Test for Differences in Selection Signatures Between Humic and Clear-Water Environments

To test whether the distribution of regions with reduced heterozygosity differed significantly between the two environments, we performed a Fisher's exact test using the *fisher.test* function in R. This test compared the observed counts of regions in each environment to the expected counts under the null hypothesis of equal proportions. A significant result indicated that heterozygosity reduction was non-randomly associated with the environmental context, suggesting potential adaptive processes.

Summary statistics for Pool Sample Sizes and Sequencing Depth in Humic vs. Clear Lakes

The humic lakes and clear water lakes showed differences in pool sample sizes and sequencing depth metrics. The average pool sample size for humic lakes was 25.05, with a median of 22.5, while clear water lakes had a slightly larger average sample size of 28.45 and a median of 28.5. Coverage also differed between the two types of lakes: humic lakes had an average coverage of 50.1 and a median of 53.3, whereas clear water lakes exhibited a lower average coverage of 46.4 and a median of 45.37.

MOLECULAR ECOLOGY

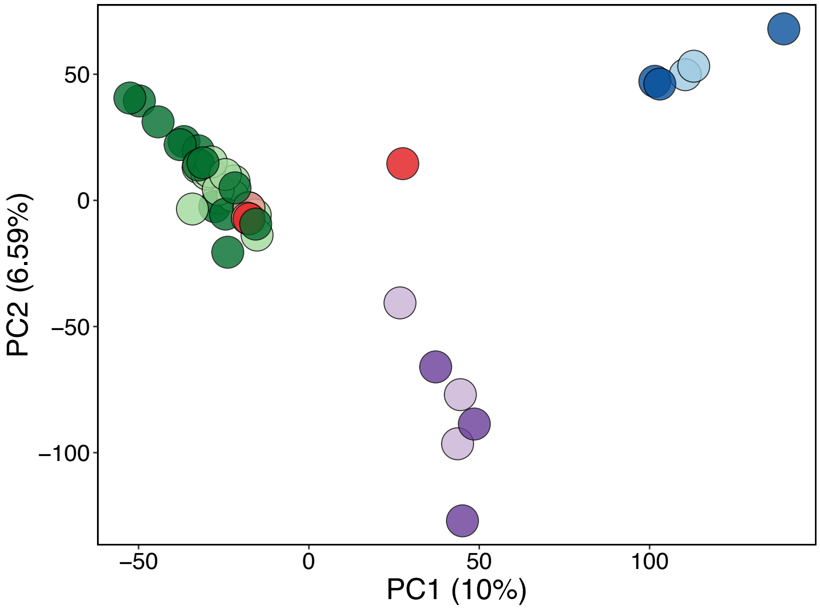

Figure S1. Principal Component Analysis (PCA) performed with 403,285 SNPs located in intergenic regions for the pool-seq dataset. Colors denote countries: greens for Estonia, blues for Finland, reds for Lithuania, and purples for Sweden.

MOLECULAR ECOLOGY

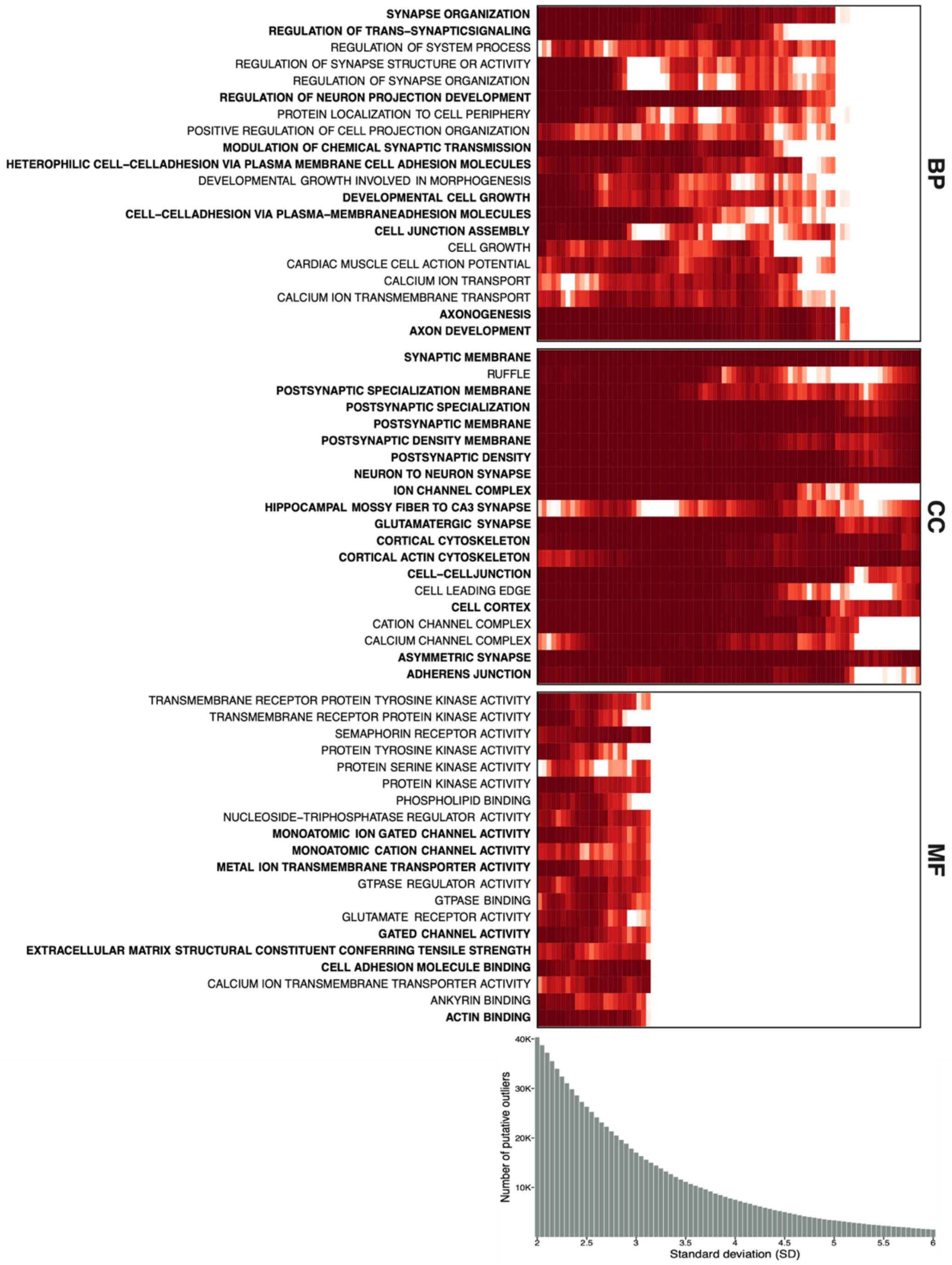

Figure S2. Dynamic outlier slicing for Gene Ontology (GO) enrichment analysis. The 20 most prevalent GO terms are plotted across the three ontologies: molecular function (MM), cellular component (CC) and biological process (BP) for the ind-seq dataset. GO terms in bold represents categories common to both pool-seq and ind-seq datasets.

MOLECULAR ECOLOGY

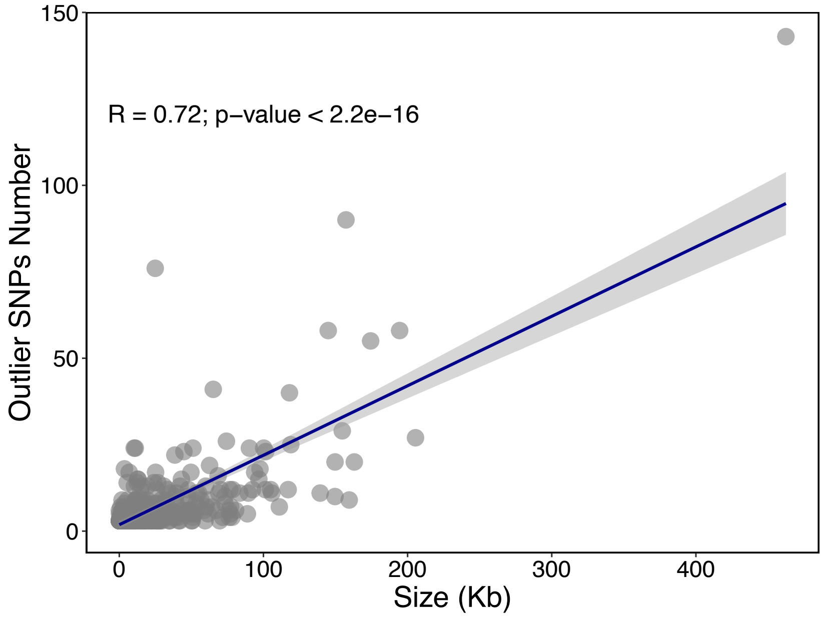

Figure S3. Correlation of size of genomic regions under selection in base pair and number of outlier SNPs within the regions.

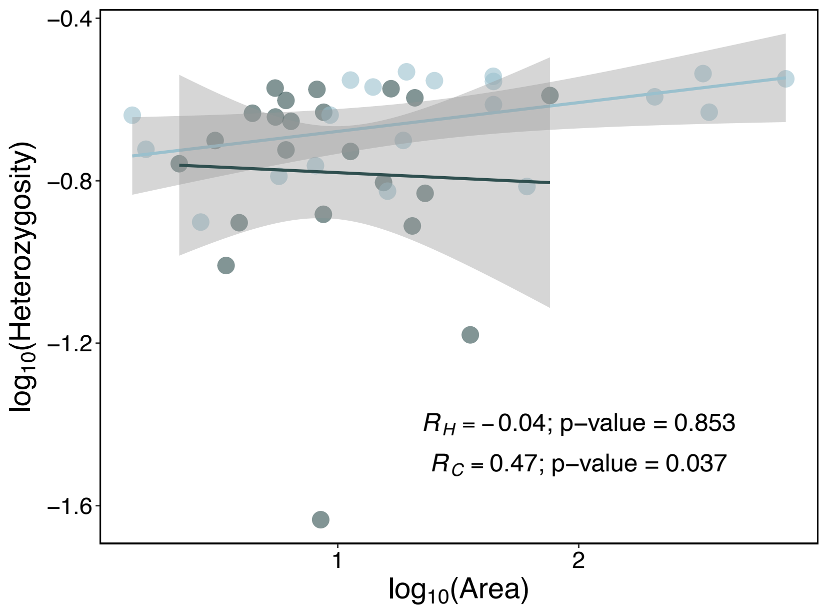

Figure S4. Correlation between heterozygosity and lake size (ha), both presented on a logarithmic scale, for humic and clear-water lakes populations. Clear lakes are represented in light blue, while humic lakes are shown in gray (including data points and regression lines). R_C denotes the correlation coefficient for clear lakes, and R_H represents the correlation coefficient for humic lakes.

MOLECULAR ECOLOGY

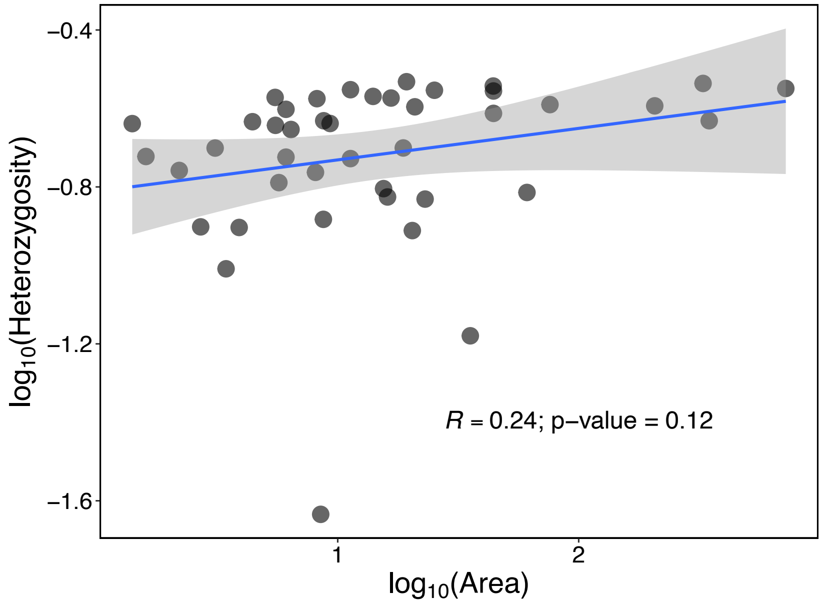

Figure S5. Correlation between heterozygosity and lake size (ha), both presented on a logarithmic scale, for all populations.

Supplemental 2. R Script for Generating Files of SNPs Passing Each Threshold

```
# Clear the workspace and load required libraries
rm(list = ls())
library(dplyr)
library(data.table)
```

1. Read input data (Results for CSS test of the complete dataset 873788 SNPs)

```
data <- read.table("CSS_results_42pools_873KSNPs.txt", header = TRUE)
head(data)
##                  SNP         CSS FDR
## 1 CM020909.1_51391 0.005778296   1
## 2 CM020909.1_60388 0.131296136   1
## 3 CM020909.1_61660 0.069444106   1
## 4 CM020909.1_61709 0.037325384   1
## 5 CM020909.1_62791 0.251888291   1
## 6 CM020909.1_62829 0.066506878   1
```

2. Generate a sequence of Standard Deviation (SD) threshold values from 2 to 6 with increments of 0.05.

```
n_sequence <- seq(2, 6, by = 0.05)
head(n_sequence)
## [1] 2.00 2.05 2.10 2.15 2.20 2.25
length(n_sequence)  # Check the number of thresholds generated
## [1] 81
```

3. Subset the data based on CSS thresholds and store results

```
# This list will hold subsetted data frames for each threshold
output_list <- list()

# Loop through each SD threshold value and subset data accordingly
for (n in n_sequence) {
  results <- data %>%
    filter(CSS > mean(data$CSS, na.rm = TRUE) + n * sd(data$CSS, na.rm = TRUE))
  # Append the subsetted results to the output list
  output_list[[length(output_list) + 1]] <- results
}
```

4. Inspect results

```r
# Check dimensions and content of the first and last subsetted data frames
dim(output_list[[1]])  # Dimensions of the first threshold result
## [1] 44974     3
dim(output_list[[length(output_list)]])  # Dimensions of the last threshold result
## [1] 1228    3
head(output_list[[1]])  # First few rows of the first threshold result
##                  SNP      CSS       FDR
## 1  CM020909.1_99311 2.631283 0.1361019
## 2 CM020909.1_406346 2.743985 0.1227968
## 3 CM020909.1_412280 1.982203 0.2478061
## 4 CM020909.1_505374 1.861864 0.2767020
## 5 CM020909.1_540819 2.015060 0.2404317
## 6 CM020909.1_540838 2.148406 0.2121403
head(output_list[[length(output_list)]])  # First few rows of the last threshold result
##                   SNP      CSS        FDR
## 1 CM020909.1_3103428 4.691175 0.01931307
## 2 CM020909.1_4784360 4.712993 0.01885152
## 3 CM020909.1_4784362 4.710322 0.01887458
## 4 CM020909.1_4796975 6.168399 0.00364394
## 5 CM020909.1_7305631 4.583675 0.02156039
## 6 CM020909.1_7858000 4.499179 0.02391465
```

5. Export subsetted results to files

```r
# Define the file name template
file_name_template <- "CSS_sd_%.2f.txt"

# Loop through the output list and export each data frame as a text file
for (i in seq_along(output_list)) {
  # Get the current data frame and its corresponding threshold value
  current_df <- output_list[[i]]
  n <- n_sequence[i]

  # Generate a file name based on the current threshold value
  file_name <- sprintf(file_name_template, n)

  # Write the data frame to a text file with tab-separated values
  write.table(current_df, file = file_name, row.names = F, col.names = T, quote = F, sep = "\t")
}
```

Supplemental 3. R Script for Calculating Enrichment and Depletion Across Different Thresholds.

```
# Clear the workspace and load required libraries
rm(list = ls())
library(dplyr)
library(janitor)
library(data.table)
```

1.Load input files

```
# 1. Read the count table of annotation for all SNPs.
count_all <- read.table("Annotation_count_table_873KSNPs.txt", header = TRUE)
head(count_all)
```

```
##                                         Category  Count Group Proportion_all
## 1                             3_prime_UTR_variant  35938   All    0.030909782
## 2 5_prime_UTR_premature_start_codon_gain_variant   1500   All    0.001290129
## 3                             5_prime_UTR_variant   8654   All    0.007443187
## 4                         downstream_gene_variant 138835   All    0.119410084
## 5                              intergenic_region 403285   All    0.346859911
## 6                                 intron_variant 380184   All    0.326991057
```

```
# 2. Read the full annotation file for all 873,788 SNPs
data0 <- read.table("Complete_annotation_poolseq.txt", header = TRUE)
head(data0)
```

```
##        CHROM START   END REF ALT                   ANN              SNP
## 1 CM020909.1  1167  1167   C   T     intergenic_region  CM020909.1_1167
## 2 CM020909.1  1292  1292   A   C     intergenic_region  CM020909.1_1292
## 3 CM020909.1  1331  1331   T   C     intergenic_region  CM020909.1_1331
## 4 CM020909.1 34877 34877   G   A     intergenic_region CM020909.1_34877
## 5 CM020909.1 51288 51288   G   A upstream_gene_variant CM020909.1_51288
## 6 CM020909.1 51288 51288   G   A     intergenic_region CM020909.1_51288
```

```
# 3. Read the list of filenames of CSS threshold files (created in the previous step)
file_names <- list.files(pattern = "CSS_sd.*\\.txt", full.names = TRUE)
```

2. Calculate SNP enrichment/depletion for each threshold

```
#Initialize an empty list to store results
results_list <- list()

# Process each file in the list
for (file_name in file_names) {
```

```r
  # Step 1: Load the CSS files with SNPs for each threshold and obtain their annotations
  data <- read.table(file_name, header = TRUE, sep = "\t")
  outlier <- data0 %>% filter(SNP %in% data$SNP)
  # Create a count table for annotation categories
  outlier_tb <- as.data.frame(table(outlier$ANN))
  colnames(outlier_tb) <- c("Category", "Count")
  # Estimate the proportion for annotation categories
  outlier_tb <- transform(outlier_tb, Group = "Outlier", Proportion_out = Count / sum(Count))

  # Step 2: Join the count table with proportion for each threshold and whole data set (count_all)
  prop_wide <- merge(count_all, outlier_tb, by.x = "Category", by.y = "Category", all.x = TRUE)
  prop_wide$Group.y[is.na(prop_wide$Group.y)] <- "Outlier"
  prop_wide[is.na(prop_wide)] <- 0
  prop_wide <- prop_wide %>% adorn_totals("row")
  prop_wide$CONDITION <- ifelse(prop_wide$Proportion_all <= prop_wide$Proportion_out,
                               "ENRICHMENT", "DEPLETION")
  prop_wide <- data.frame(prop_wide)

  # Step 3: Perform chi-square tests for each category
  rchi <- list()
  for (i in 1:14) {
    rchi[[i]] <- chisq.test(prop_wide[c(i, 15), c(2, 5)], correct = TRUE)
  }

  # Step 4: Extract chi-square statistics and p-values
  chis <- list()
  pvalues <- list()
  for (i in 1:14) {
    chis[[i]] <- unname(rchi[[i]][[1]])
    pvalues[[i]] <- unname(rchi[[i]][[3]])
  }

  # Step 5: Combine results into a data frame
  c <- cbind(data.frame(unlist(chis, use.names = TRUE)),
             data.frame(unlist(pvalues, use.names = TRUE)))
  d <- data.frame(prop_wide[1:14, ], c)

  # Step 6: Rename the last columns
  names(d)[c(9, 10)] <- c("X_squared", "p_value")

  # Step 7: Finalize the output format and add SD and SNP count information
  d1 <- d[, c(1, 4, 7, 8, 9, 10)]
  d1$SD <- gsub("CSS_sd_|\\.txt$", "", basename(file_name))
  d1$nsnps <- nrow(data)

  # Store the result in the list
  results_list[[file_name]] <- d1
}
```

3. Inspect results

```r
head(prop_wide) # how prop_wide looks like
```

```
##                                        Category Count.x Group.x Proportion_all
## 1                            3_prime_UTR_variant   35938     All    0.030909782
## 2 5_prime_UTR_premature_start_codon_gain_variant    1500     All    0.001290129
## 3                            5_prime_UTR_variant    8654     All    0.007443187
## 4                        downstream_gene_variant  138835     All    0.119410084
## 5                              intergenic_region  403285     All    0.346859911
## 6                                 intron_variant  380184     All    0.326991057
##   Count.y Group.y Proportion_out  CONDITION
## 1      61 Outlier   0.0360307147 ENRICHMENT
## 2       1 Outlier   0.0005906675  DEPLETION
## 3      20 Outlier   0.0118133491 ENRICHMENT
## 4     236 Outlier   0.1393975192 ENRICHMENT
## 5     564 Outlier   0.3331364442  DEPLETION
## 6     498 Outlier   0.2941523922  DEPLETION
```

```r
head(results_list[[1]])
```

```
##                                        Category Proportion_all Proportion_out
## 1                            3_prime_UTR_variant    0.030909782    0.033050833
## 2 5_prime_UTR_premature_start_codon_gain_variant    0.001290129    0.001348674
## 3                            5_prime_UTR_variant    0.007443187    0.007642485
## 4                        downstream_gene_variant    0.119410084    0.121397293
## 5                              intergenic_region    0.346859911    0.339882449
## 6                                 intron_variant    0.326991057    0.331440750
##    CONDITION X_squared     p_value   SD nsnps
## 1 ENRICHMENT 8.0939183 0.004441404 2.00 44974
## 2 ENRICHMENT 0.1090804 0.741193523 2.00 44974
## 3 ENRICHMENT 0.2751017 0.599929864 2.00 44974
## 4 ENRICHMENT 1.6589143 0.197749983 2.00 44974
## 5  DEPLETION 5.9880540 0.014403083 2.00 44974
## 6 ENRICHMENT 2.5681871 0.109032619 2.00 44974
```

4. Export results

```r
# Unlist the results to make a single data.frame
combined_df <- do.call(rbind, results_list)

# Arrange the combined data frame by SD value
combined_df1 <- combined_df %>% arrange(as.numeric(SD))
rownames(combined_df1)<-NULL
head(combined_df1)
```

```
##                                        Category Proportion_all Proportion_out
## 1                            3_prime_UTR_variant    0.030909782    0.033050833
## 2 5_prime_UTR_premature_start_codon_gain_variant    0.001290129    0.001348674
```

```
## 3                            5_prime_UTR_variant    0.007443187    0.007642485
## 4                        downstream_gene_variant    0.119410084    0.121397293
## 5                              intergenic_region    0.346859911    0.339882449
## 6                                 intron_variant    0.326991057    0.331440750
##    CONDITION X_squared     p_value   SD nsnps
## 1 ENRICHMENT 8.0939183 0.004441404 2.00 44974
## 2 ENRICHMENT 0.1090804 0.741193523 2.00 44974
## 3 ENRICHMENT 0.2751017 0.599929864 2.00 44974
## 4 ENRICHMENT 1.6589143 0.197749983 2.00 44974
## 5  DEPLETION 5.9880540 0.014403083 2.00 44974
## 6 ENRICHMENT 2.5681871 0.109032619 2.00 44974
```

```r
# Write the final output to a text file
write.table(combined_df1, "pvalues_sd2-8.txt", row.names = FALSE,
            col.names = TRUE, quote = FALSE, sep = "\t")
```
